# Beyond private 5G networks: applications, architectures, operator models and technological enablers

**DOI:** 10.1186/s13638-021-02067-2

**Published:** 2021-12-04

**Authors:** Mickael Maman, Emilio Calvanese-Strinati, Lam Ngoc Dinh, Thomas Haustein, Wilhelm Keusgen, Sven Wittig, Mathis Schmieder, Sergio Barbarossa, Mattia Merluzzi, Francesca Costanzo, Stefania Sardellitti, Henrik Klessig, Savita Vitthalrao Kendre, Daniele Munaretto, Marco Centenaro, Nicola di Pietro, Shuo-Peng Liang, Kuan-Yi Chih, Jack Shi-Jie Luo, Ling-Chih Kao, Jiun-Cheng Huang, Jen-Sheng Huang, Tzu-Ya Wang

**Affiliations:** 1 Univ. Grenoble Alpes, CEA, Leti, Grenoble, France; 2 Fraunhofer Heinrich Hertz Institute, Berlin, Germany; 3 Sapienza University of Rome, Rome, Italy; 4 Robert Bosch GmbH, Renningen, Germany; 5 Athonet Srl, Bolzano Vicentino, Italy; 6 ITRI, Hsinchu, Taiwan; 7 Chunghwa Telecom, Taipei, Taiwan; 8 Alpha Networks Inc., Hsinchu, Taiwan; 9 Institute for Information Industry, Taipei, Taiwan

**Keywords:** Beyond-5G, Private networks, Operator models, Application requirements

## Abstract

Private networks will play a key role in 5G and beyond to enable smart factories with the required better deployment, operation and flexible usage of available resource and infrastructure. 5G private networks will offer a lean and agile solution to effectively deploy and operate services with stringent and heterogeneous constraints in terms of reliability, latency, re-configurability and re-deployment of resources as well as issues related to governance and ownership of 5G components, and elements. In this paper, we present a novel approach to operator models, specifically targeting 5G and beyond private networks. We apply the proposed operator models to different network architecture options and to a selection of relevant use cases offering mixed private–public network operator governance and ownership. Moreover, several key enabling technologies have been identified for 5G private networks. Before the deployment, stakeholders should consider spectrum allocation and on-site channel measurements in order to fully understand the propagation characteristic of a given environment and to set up end-to-end system parameters. During the deployment, a monitoring tools will support to validate the deployment and to make sure that the end-to-end system meet the target KPI. Finally, some optimization can be made individually for service placement, network slicing and orchestration or jointly at radio access, multi-access edge computing or core network level.

## Introduction

The fifth generation of mobile communication networks (5G) expands the scope of communication wireless networks beyond individual human end users toward an integrated communication system, which also provides wireless connectivity to new vertical applications driven by industries such as manufacturing, automotive, health or agriculture. While 4G was largely associated with traditional operator models, due to nationwide spectrum allocation and tremendous infrastructure costs, 5G opens a new market opportunity for private networks and early 6G research [1] anticipates that private networks will play a fundamental role in further evolutions of cellular networks. 5G and beyond future private networks are key enabler for future smart factories with the required better deployment, operation and flexible usage of available resource and infrastructure. 5G private networks will offer a lean and agile solution to effectively deploy and operate services with stringent and heterogeneous constraints in terms of reliability, connect-compute latency, re-configurability and re-deployment of resources as well as issues related to governance and ownership of 5G components, and elements. To this end, several key enabling technologies have been already identified and integrated for 5G public networks such as network slicing and orchestration, Multi-Access Edge Computing (MEC) support and machine learning at the edge support. Nevertheless, private networks impose specific context-dependent challenges with respect to local ownership, governance and optimization of locally deployed resources. This includes dynamic configuration and reconfiguration of hardware and software components to determine which part of the network belongs to private or public operators.

In the literature, there is a growing interest in non-public network [2], private network [3–8], campus network [9], micro-licensing for locally operated networks [10], neutral host networks [11] or private unlicensed networks [12]. Larmo et al. [3] propose private 5G networks to address critical wireless communication requirements (i.e., reliability, availability, quality of service, security and interworking) in public safety, infrastructure and industry. Ordonez et al. [4] and 5G-ACIA alliance [2] provide an overview of 5G non-public networks, studying their applicability to the industry 4.0 ecosystem. They identify a number of deployment options relevant for non-public networks, and discuss their integration with Mobile Network Operators (MNO) public networks according to different criteria, including technical, regulatory and business aspects. Brown [5] investigates key issues in private 5G networks, including how the end-user can deploy and operate the technology, the integration of private local-area and wide-area public networks, and the importance of spectrum for private deployments. In particular, he discusses how private 5G can be deployed across diverse licensed, shared licensed, and unlicensed spectrum bands. Rostami [6] analyzes, classifies and compares, with a comprehensive set of evaluation metrics, the potential deployment architecture and operation models for private 5G networks. He shows that each model comes with its own benefits and costs, and that not all the quality metrics can be optimized at the same time. Thus, it is crucial to analyze the usage scenarios and identify corresponding requirements before choosing a suitable operation and deployment model. Aijaz [8] provides an overview of several technical aspects of private 5G networks for industrial applications and scenarios. In particular, he discusses their functional architectures, use cases, spectrum opportunities, and advanced features, like network slicing, time-sensitive networking, and optimization of resource allocation. It also provides a few overall considerations on the related business opportunities. Matinmikko et al. [10] propose to deploy local radio access networks and offer dedicated vertical services in specific areas with micro-licensing allowing different stakeholders to use 5G spectrum locally on a shared basis. They identify the regulatory implications when the 5G end-to-end network spans across different stakeholders administrative domains and seek to address how spectrum authorization should be done for the future 5G networks. They present the local micro-licensing model from regulatory perspective to complement the existing individual authorization and general authorization models by combining their benefits. Siriwardhana et al. [13] focuses on localized 5G networks and local operators that operate such networks within their premises with guaranteed quality and reliability to complement MNOs offerings. The authors investigate specific network architectures (and the related network function deployments), with a focus on two use cases: massive wireless sensor networks and mobile robots. Ahokangas et al. [7] examine a complex industrial stakeholder ecosystem and build a private 5G-enabled platform ecosystem (i.e., data and connectivity platform configurations) to benefit from digitalization and serve its stakeholders. They also identify the regulatory challenges of deploying private 5G networks (e.g., spectrum, operator role, competition, access to infrastructure, radio equipment authorization, security and privacy, and vertical specific regulation). Bajracharya et al. [12] argue that the unlicensed spectrum plays a crucial role in private network but faces various limitations and restrictions (i.e., region and band specific, reduced efficiency) due to the coexistence of networks in the shared band. They discuss about potential solutions such as listen before talk with maximum channel occupancy time, adaptive back-off, handover skipping, self-organized network and multi-domain coexistence. Kibria et al. [11] introduce a new business model called neutral host micro-operator networks in shared spectrum. This self-contained network allows authentication of SIM-based or SIM-less access to provide localized and local context-based services by location owners for subscribers of different service providers and satisfying coverage extension and traffic offload.

Several papers in the literature address operators’ Business Models (BM) for building the right ecosystem for 5G. Rao et al. [14] discuss the impact of 5G technologies on BMs and how telecom operators differentiate their businesses (e.g., connectivity provider, third-party partnership and digital service provider). Kuklinski et al. [15] analyze the BMs enabled by network slicing. They identified the distinct roles of key players and their business interactions, particularly for multiple infrastructure owners and service providers. Camps-Arago et al. [16] also analyze BMs with the adoption of 5G and network slicing technologies and discuss six potential models: (i) micro-operator that does not own spectrum and only operates in a specific area, (ii) provider of Xaas offering something as a service, (iii) use case enabler addressing business to business relationships, (iv) ecosystem orchestrator playing the structural role of leading efforts to foster relationships beyond its direct customers, (v) pervasive platforms striving to dominate the value chain and extract substantial rents, and (vi) specific ecosystem within their physical boundaries. Ahokangas et al. [17] explore other generic BMs for local 5G micro-operators, including vertical (i.e., based on use case similarity), horizontal (i.e., based on direct business to business in localized domain) and oblique (i.e., based on a platform that supports mass-tailoring). Sacoto-Cabrera et al. [18] implement two BMs: strategic (e.g., when the MNO and the Virtual Operator (VO) provide service to its own user bases and the MNO receives revenue per VO subscriber) and monopolistic (e.g., when the MNO provides service to both user bases). Noll et al. [19] introduce collaborative BMs based on the postulation of 5G as a system of systems. Collaborative BMs are known by the sharing of network infrastructure and when mobile and indoor operator have agreements to share revenues. Hmoud et al. [20] study BM platformization and big data-driven BM with two or more parties interacting with each other and propose a two-sided advertising BM using design science approach and grounded theory principles.

In this paper, we present the vision and first results of the 5G CONNI joint Europe-Taiwan collaborative H2020 project [21]. Its activities revolve around the use of non-public 5G networks in Industry 4.0 applications, ultimately aiming at deploying a 5G end-to-end testing system for key enabling technologies ranging from 5G Core, MEC and 5G Radio Access Network (RAN) to the industrial use cases. As GSMA [22] for 5G standalone network deployment, we aim to provide a detailed guideline for the deployment of private 5G networks. For the 5G CONNI demonstration system, three challenging industrial manufacturing use cases are presented accompanied by a number of use case scenarios for multi-site connectivity.

5G private networks along with technical enhancements revolving around softwarization, cloudification and increased modularity of the 5G System are expected to disrupt the current constellation not only regarding deployment models and architectures but also with respect to the stakeholders involved in the operation of a private network and their roles and responsibilities, i.e., operator model. Anticipating initial concerns by different stakeholders to novel operator models, it is important to explore this field unbiased, in order to better understand the different degrees of freedom of operator models and how they are interrelated. 5G elements (e.g., 5G network functions, RAN components, etc.) and non-5G elements (e.g., enterprise Information Technology (IT)), private 5G network lifecycle tasks and involved stakeholders (e.g., enterprise, MNO, service provider) are important and interrelated dimensions. For example, whether one stakeholder can carry out management task on a 5G component depends on a multitude of factors, including the physical and logical location of that element (e.g., 5G Core) and on what other stakeholders own and govern. In this paper, we provide an in-depth analysis of operator models, associated requirements and concerns to be addressed. In general, such concerns to novel operator models are attributed to different perspectives: confidentiality, integrity and availability of information, access to and control of elements (specifically 5G components), the private 5G network life-cycle and responsibilities, business models and expertise required by the stakeholders for each task, regulatory aspects, and applicability and practicability.

The success of private 5G network operations strongly depends on their architecture and deployment strategies. In this paper, we therefore present novel network architectures, namely: (i) fully private, more suitable for Industry 4.0; (ii) Mobile Virtual Network Operator (MVNO), in which the RAN is shared across private and public network; (iii) hybrid model, which is a combination of the above solutions; (iv) MNO’s private core network, in which core/transport networks, spectrum and Subscriber Identity Module (SIM) cards are own by the operator. The interdependence of operator and network deployment models is analyzed, as well as its dependence on the ownership of network elements.

**Table 1:** Non-functional requirements

Non-functional requirement	UC-1	UC-2	UC-3
Service bitrate	208 Mbps per machine	Up to 1 Gbps per device	0.2–1.6 Mbps (control), 100s Mbps (video)
Communication area	Some 1000 $\hbox {m}^{2}$ (2644 $\hbox {m}^{2}$ for demo setup)	Some 1000 $\hbox {m}^{2}$ (2644 $\hbox {m}^{2}$)	100 m × 100 m × 15 m
Connection density	10s per shop floor (11)	Up to 10 per shop floor (6)	1 to few tens per shop floor
Area traffic capacity	86.5 Mbps per 100 $\hbox {m}^{2}$	227 Mbps per 100 $\hbox {m}^{2}$	100s Mbps per 100 $\hbox {m}^{2}$
UE speed	stationary	< 3 km/h	< 2 km/h
Positioning acc.	n/a	< 1 m (horiz.)	n/a
Positioning lat.	n/a	< 15 ms	n/a
Motion-to-photon latency	n/a	< 50 ms	n/a
End-to-end latency	n/a	< 10 ms	1–7 ms
Transfer interval	n/a	n/a	5–20 ms
Transmission time	n/a	n/a	1.4–7 ms
Survival time	n/a	n/a	20 ms
Message size	n/a	n/a	200 bytes
Video latency	n/a	n/a	< *N* times transf. int.

Finally, an important aspect of private networks pertains the technological enablers, which include technological components and optimization methodologies. In this regard, we present a detailed description of spectrum allocation and channel models, with a specific original investigation based on a measurement campaign performed in a factory. Channel models are essential for planning the deployment of access points and sensor nodes, in order to fulfill the network requirements. Once the network has been deployed and is ready for operations, network monitoring is required to assess the performance of the deployment. Therefore, novel network monitoring strategies are described, with the aim of improving network behavior. From an application point of view, services deployment (i.e., service placement) is analyzed and novel strategies are presented with numerical results to assess the performance in industrial scenarios. Last but not least, an overview of different key enabling technologies is presented, with novel proposal of MEC, network slicing, orchestration and core network implementations. Furthermore, a joint optimization of the enabling technologies is proposed. Numerical results, performed with industrial channel models, assess the performance of the proposed approaches.

The paper is organized as follows. Section 2 analyzes the 5G and beyond private network use-cases selected in 5G CONNI, and Sect. 3 gives an overview on standardization aspects. Section 4 defines operator models, requirements and concerns. Section 5 presents the different architecture options and their relations with operator models. Section 6 covers the problems and optimizations related to the private 5G network deployment, and Sect. 7 concludes the paper.

## 5G and beyond private network use cases: from requirements to proof of concept

This section investigates the three use cases selected for implementation at two trial sites in Europe and Taiwan in the 5G CONNI context of private 5G networks : *Process diagnostics by Computer Numerical Control (CNC) and sensing data collection* (UC-1), *Process diagnostics using virtual or augmented reality* (UC-2) and *Robot platform with edge intelligence and control* (UC-3). For each use case, some implementation aspects and challenges are described for the demonstration implementations and studies. The analysis of non-functional and functional requirements is summarized in Tables 1 and 2, respectively.

**Table 2:** Functional requirements

Functional requirement	UC-1	UC-2	UC-3
Mobility management		X	(X)
Energy efficiency		X	
End-to-end QoS	X	X	X
Network capability exposure		X	X
Priority, QoS and policy control			X
Time synchronization		X	X
Localization service		X	
Context-aware network			X
Real-time end-to-end QoS monitoring	X	X	X
5G LAN-type service support			(X)
Proximity services			(X)
Secure remote access	X	X	(X)
Edge computing integration	X	X	X

**Fig. 1:**
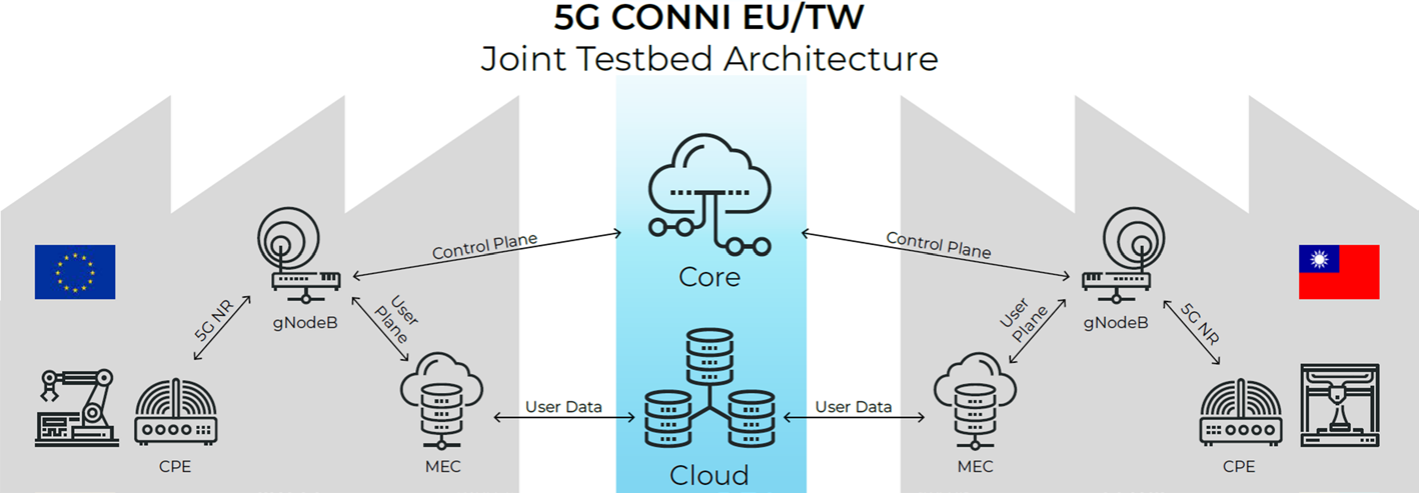
Testbed architecture of 5G CONNI

The two production facilities are each equipped with their own RAN and MEC. The user data plane is terminated locally with appropriate network functions being present in the local edge cloud. As productive real-world industrial manufacturing may be distributed over multiple facilities and large geographical distances, an approach for interconnecting sites via wide area networks is considered as shown in Fig. 1. Here, the control plane of the network resides at a central location (e.g., within a public cloud environment), allowing mobility of equipment, assets and data.

**Fig. 2:**
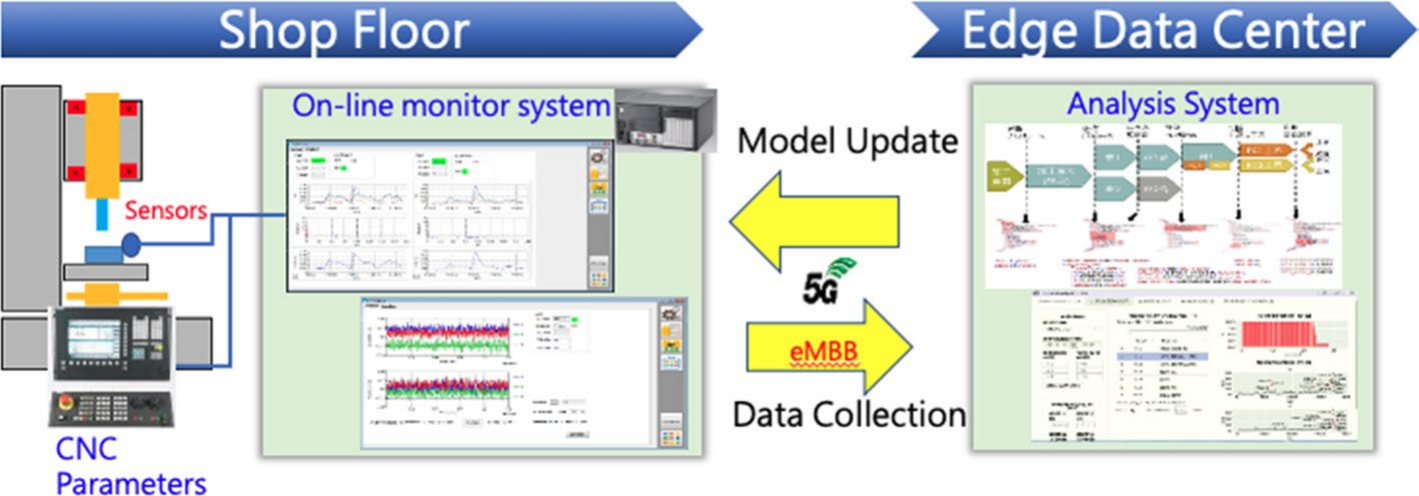
Implementation of process diagnostics by CNC and sensing data collection

The manufacturing shop floor is an environment typically characterized by high operational, security and safety standards. In this regard, the target proof of concept will consider some important restrictions, regulations and challenges, which can be summarized as follows: (1) planning, design and roll-out of the 5G network without disturbing the ongoing production tasks, (2) 5G system integration without disturbing the existing networking system, (3) ensuring safety for factory personnel, (4) considering special propagation characteristics due to the shop floor environment, (5) enterprise IT security regulations, for example, to securely provide a service provider access to a corporate network, and (6) factory IT security, in particular, the concept of security zones, as introduced in ISA/IEC 62443 [23].

### UC-1: process diagnostics by CNC and sensing data collection

To meet the requirements of small batch production and massive customization, configuration of machine tools or production stations needs to be flexible. The architecture of conventional CNC systems for machine tools is fixed. Hence, once the configuration and number of axes is decided, the software architecture and control block diagram are fixed. However, in order to meet the massive customization scenario, the number of moving axes and combination of hybrid manufacturing processes (e.g., additive and machining processes) must be able to be modified with very low cost to quickly respond to many small batch or one piece contracts. Although the industrial control over 5G network or cyber-physical control has been described in [24] and communications for automation in vertical domains, such as factory of the future has been described in [25], design, implementation and deployment of a cyber-physical controller is still needed to define actual specification for various scenarios (e.g., flexible machining machine consisting of many spindles and moving axes to perform sequential operations of a specific part, or a specialized production station consisting of an additive 3D printing machine and subtractive milling or turning machines).

In UC1, various sensors will be attached on the spindle and workpiece with sampling rates between 10 and 400 kHz to collect all necessary physical quantities. As shown in Fig. 2, collected data will be aggregated into an online monitor system and transferred via 5G to the analysis system, which is deployed in the edge data center to conduct process diagnosis. Updated model parameters and threshold values derived from the analysis system are transferred back to the online monitor system to monitor the milling process in real time.

**Fig. 3:**
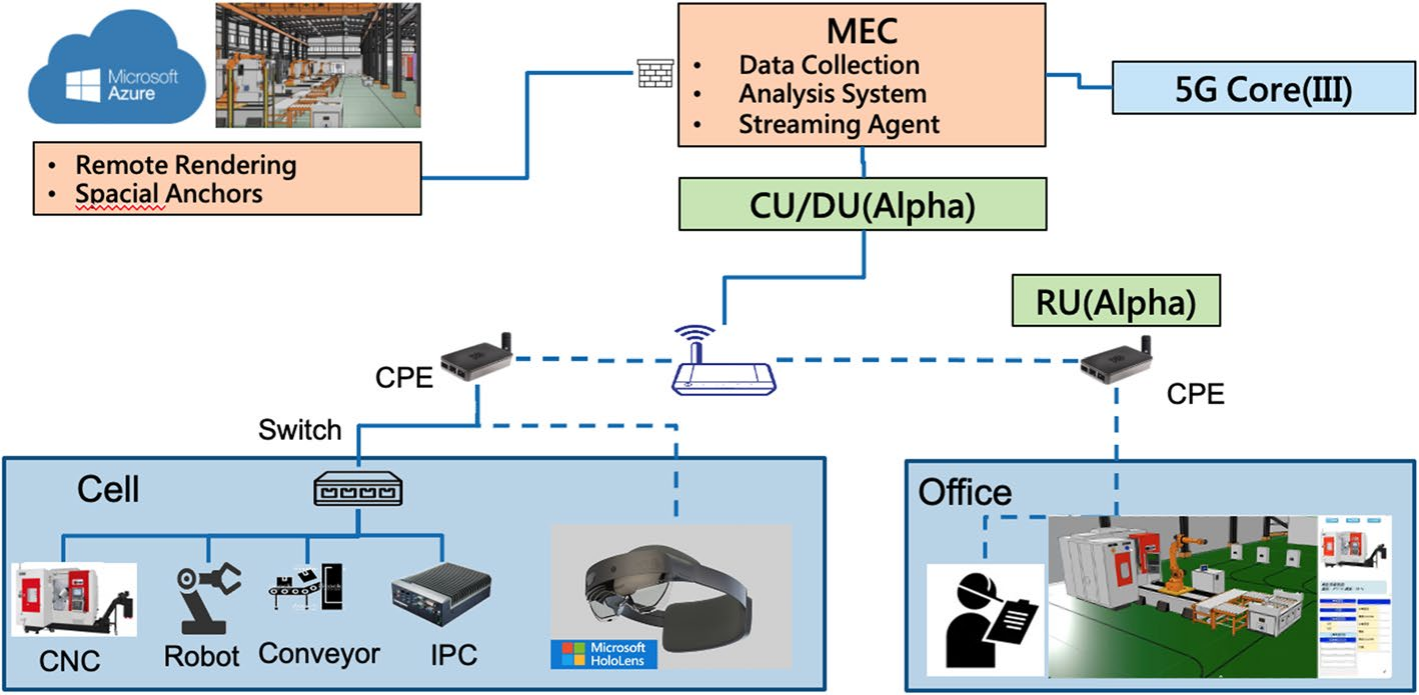
Implementation of AR/VR for process diagnostics

First, a target cell (i.e., CNC lathe, a robot and a conveyor) will be studied. This equipment will be connected to an industrial PC running the online monitor system. The collected data will be uploaded to the analysis system in an edge data center using the 5G CPE and gNB. A detailed analysis of the requirements will be performed. Lastly, the setup will be extended to 11 CNC cells distributed throughout the shop floor, and a network emulator will introduce different impairments (bandwidth limitations, latency and packet loss) to assess the impact on the applications.

### UC-2: process diagnostics using augmented/virtual reality

Conventional machining process planning relies on time-consuming Trial-and-Error (T&E) activities to determine force-related parameters (e.g., feed rate, spindle speed, depth and width of cut). In order to reduce the T&E cost, additional diagnosis models are used to detect vibration, collision, acoustic emission, temperature, or energy consumption—input that goes beyond that of traditional computer-aided manufacturing software to generate tool paths along a workpiece geometry. Virtual or Augmented Reality (VR/AR) can help process engineers reveal full productivity of machines by superimposing graphical objects (e.g., 3D models, charts, vector fields and text messages) on top of a video streaming on head-mounted displays. Here, high data rate along with low latency properties of 5G can be utilized, while high fidelity 3D scenes with millions of polygons are rendered remotely and transferred wirelessly lightweight VR/AR device in terms of video streams to facilitate high mobility on the shop floor. Recognized objects (e.g., machines, spindles and workpiece) are then linked with the digital twins to query corresponding 3D models, sensing data, and condition values, and synthesized as virtual objects in the VR/AR scenes. For example, tool paths can be plotted and color-coded according to features, such as the vibration level, and superimposed on the real machine image or the machine 3D model. This enables process engineers to observe machining conditions in a more intuitive way, shorten the T&E process planning time and interact with various digital twins at the same time in a focused and hands-free manner.

Figure 3 illustrates the architecture of the AR/VR use case. The 5G network will be deployed on the shop floor to connect equipment (e.g., CNC, robot, conveyor and AGV) with a CPE to link with the gNB and the edge cloud. The data collection is achieved by a secured open platform communications unified architecture client server pair. The control agent is a software module located in the edge data center. The agent, implemented by the Unity3D software, will act as a bridge between user and 3D scene. In this setup, the user device will be a tablet PC or head-mounted display with a camera module.

**Fig. 4:**
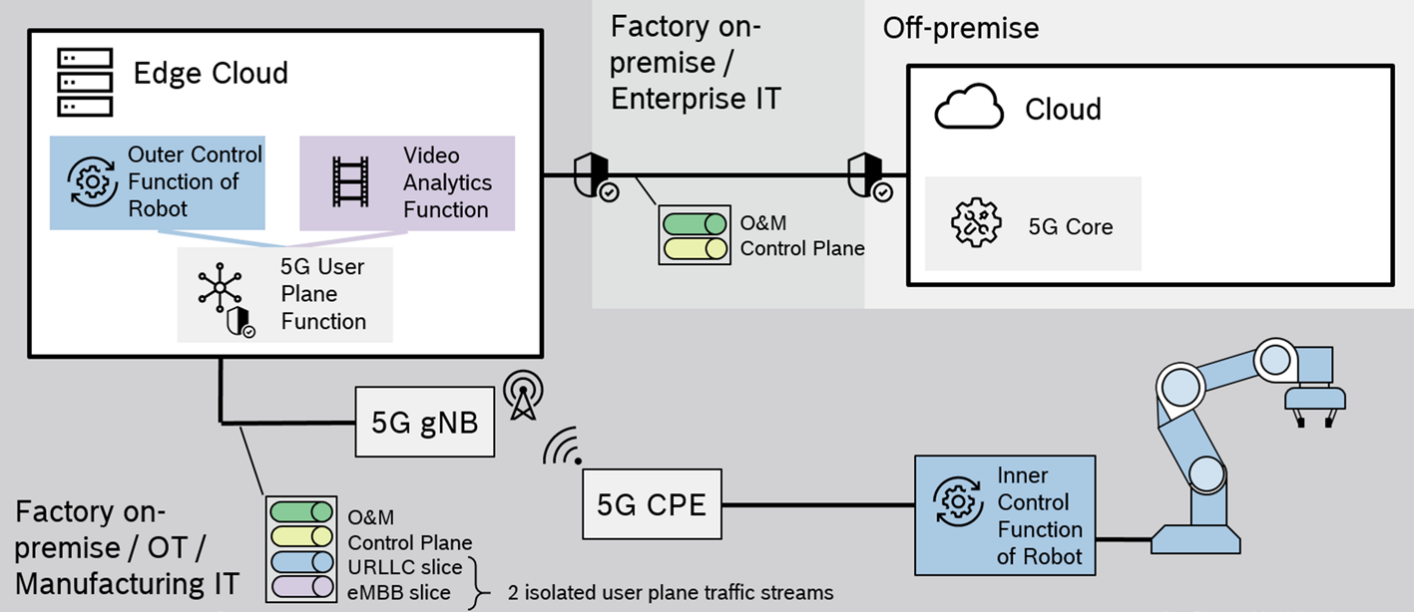
Implementation of robot platform into factory IT

Using the camera module, the relative position and orientation between the end user and the equipment can be identified and transformed into corresponding viewport and navigation commands to the 3D model. Sensing data can be associated with corresponding 3D components to show process status information. For example, vibration levels can be shown on the 3D model of the spindle as color-coded contour. Machine users will use the plotted tool paths and other 3D objects, such as charts or text messages, to optimize the machining parameters. Performance between users using the conventional T&E method and users with AR/VR devices will be compared in terms of total process planning time, cycle time for the workpiece before and after optimization.

### UC-3: robot platform with edge intelligence and control

Robot platforms can perform assembly, inspection, packaging, and other complex tasks, but their flexibility is limited when stationary. However, processing smaller batches for increased product variety demands for greater flexibility (mobile robots), while at the same time production performance needs to be improved, e.g., in terms of overall equipment efficiency. One approach to achieve this is to offload (parts of) the control functions to the local edge cloud (edge programmable logic controllers), which would not only improve efficiency, but also unlock more synergistic optimization potential through the pooling of computing resources. Besides, production efficiency can be improved by centrally managing, troubleshooting, monitoring and programming of remote robot and machine controllers in the cloud. A 5G link can interconnect the robot with the backend to fulfill the speed and reliability requirements imposed by the exchange of motion control messages (e.g., target values of joint positions or speeds) and feedback (e.g., joint angles and torques).

In our planned setup (cf. Fig. 4), we use a 7-degree-of-freedom Franka Emika’s collaborative robotic arm called Panda to demonstrate the performance of the underlying 5G network between the edge controller and the robot. The Panda robot is normally connected to a workstation PC via Ethernet and requires communication to the controller at 1 kHz. Franka uses a real-time robot control code using UDP messages via the Franka Emika interface. This interface includes a motion generator and a controller, through which a programmer can send commands to the robot. Motion generator defines robot motion in joint or coordinate space and uses an internal controller to follow the commanded motion, whereas the external controller sends torque commands directly to the servo motor joints by ignoring the motion generator. Franka can use both motion generator and external control interface also.

To demonstrate the 5G network performance of closed control loop of the Panda robotic arm, a dynamic split of the control algorithm between the edge cloud and the robot side will be implemented. The network’s Key Performance Indicators (KPI) will be analyzed, and an adaptive approach to the static control loop will be developed by taking into account the dynamic latency of the 5G network. Initially, an emulated network delay model (e.g., using a 5G network simulator) will be inserted between the control loop split parts, and the control function parameters will be modified to avoid intrinsic safety condition due to network delay by respecting the desired trajectory precision and increasing the processing time. For example, predictions about the network delay can be provided by smart machine learning-based algorithms using 5G network data analysis function. At a later stage, it is expected that the network emulator and the wired link will be replaced by the 5G system, allowing the performance of the real 5G system to be tested and using real performance metrics.

### Multi-site use cases

In addition to the individual use cases, several use cases can be realized across multiple locations. Such locations can be either multiple site of the same enterprise or sites of a many different enterprises.

#### Cross-border, intra-enterprise monitoring

Most often, large enterprises have more than one site. These sites include headquarters, office buildings and, for the industrial sector, manufacturing sites or plants, and they are interconnected with specific sets of IT rules and regulations. Manufacturing sites, in particular, can exchange information with each other in a secure way. Depending on the specific use case, this information can be process data that helps improve production processes across sites or other information that should be gathered centrally. Other information that is shared among different interconnected sites including corporate or division headquarters is data with respect to production efficiency, material consumption or overall equipment efficiency. Besides, IT systems including communication infrastructure are often managed centrally by IT experts to reduce complexity and management effort. For private 5G networks, the management and core functionalities of 5G ideally remain in a central location where the use case requirements are met. Therefore, such a scenario would require a solution in which the 5G Core is installed at the division or corporate headquarters, managed either by company-internal experts or by an external MNO or solution provider.

#### Cross-border or intra/inter-enterprise shipping of assets or production lines

Production assets, such as robots, machines, tools or workpiece carriers, are actively used in production for several years until they are replaced by other components. Nevertheless, these assets are reused in different locations, either in plants of the same enterprise or in different enterprises. Usually, after their years of production, machines and production lines are disassembled, shipped to another site and then re-assembled to produce similar or other goods. In this case, it would be advantageous to retain the wireless configurations, such as subscriber and Quality of Service (QoS) profiles that are actually tailored to the specific production line, but in the case where the production line is re-assembled at a new location, the subscriber profiles can actually remain active with the network configuration, e.g., in terms of network slices. In such a case, a unified 5G management or even 5G core would be preferred, so that the production assets work the same way at the new location as they did at the old one, while minimizing manual re-configuration of the wireless system including UEs.

Favorable solutions in this scenario would require the 5G Core to be located in the enterprise domain for central management or in a central cloud with unified management across multiple companies. Alternatively, multiple interconnected private 5G Cores that share a common database of user profile could be used.

#### Intra-enterprise or inter-enterprise lineside delivery and tracking on logistics routes

Inbound and internal logistics are important aspects of all factories, where ideally automated processes for tracking and registration of all kinds of assets and materials are applied. 5G networks will play a crucial role in positioning and identifying connected UEs in different factories, whether they belong to the same or different enterprises, as well as the logistics routes between them. While private networks cover the area in and around factories, the public 5G network provides connectivity and tracking capabilities along logistics routes. In addition to tracking assets and goods, this avoids manual inbound registration processes and leads to minimal human intervention or the use of other technologies such as RFID gates.

In this scenario, sensitive user and location data must be securely exchanged between end devices in the respective private networks, but also over the public network. In this case, roaming architectures and private communications are important elements of such a constellation.

#### Remote commissioning of machines

5G-interconnected plants and enterprises offer new opportunities for machine builders, process engineers and similar experts through innovative connectivity applications. Here, experts can remotely and securely log into machines and other assets, configure them and optimize production processes, for instance. This is not only a flexible approach but can also save effort and costs for the required personnel.

The use of remote access, maintenance, commissioning and other services requires private and highly secured connections between the expert and the machine in question. Communication must be isolated, at least logically, from other communication or data flows.

#### Remote expert support for process diagnosis

Deploying manufacturing sites overseas is a common practice for companies seeking to reduce production and logistics costs. Collaborating engineers from different location and transferring expertise to production sites results in significant traveling costs. With the COVID 19 pandemic, things got worse as almost all international travel was cancelled. 5G technology brings new opportunity to solve the above difficulty, as we can connect engineers to digital twins and interact with each other via immersive interaction devices such as AR/VR so that they can design, plan and troubleshoot in the same virtualized 3D scene. In doing so, the enterprise headquarters experts can support manufacturing sites all around the world. Thus, they reduce travel costs for collaboration and quickly deploy new manufacturing sites while keeping core technology within the enterprise and providing the necessary support with digital twins in the cloud platform.

## Standardization perspective

Standardization is another success factor for 5G private networks. 5G CONNI is devoted to a number of targeted, interrelated and closely coordinated standardization activities, mainly on 3GPP. These activities are derived from the architecture aspects as well as the research pillars. At the 3GPP RAN#80 plenary [26], several Rel-17 study and work items were agreed to address open issues in Non-Public Networks (NPN) closely related to research in 5G CONNI. The ongoing Rel-17 work item on management of Non-Public Networks [27] is an example of work defining management requirements and roles in NPN and specifying deployment scenarios, including in factories. Special attention is given to provisioning and exposure of management functions, services and data. The 5G CONNI work is well aligned with these topics and partners will actively follow and contribute to the standardization. Another ongoing Rel-17 study item is Study on Enhanced Support of NPN [28]. This study focuses on the credential and subscription handling in NPN. The onboarding and provisioning procedures will be defined, and the entities handling the subscription will be specified. Furthermore, the study aims to investigate enhancements to the service requirements for audio-visual content. These topics are very relevant to 5G CONNI and contributions to 3GPP are planned.

Several other ongoing study and work items regarding unlicensed access, dynamic spectrum sharing, edge applications and flexible local area data networks are related to NPN, highlighting the relevance of these topics that is reflected in the standardization.

## Methods to define operator model for private 5G networks

5G NPNs as well as technical enhancements around softwarization, cloudification and increased modularity of the 5G System are expected to disrupt the current telecommunication ecosystem, not only regarding deployment models and architectures but also with respect to the stakeholders involved in the private network operations and their roles and responsibilities. In other words, the rise of non-public networks calls for the definition of new operator models, taking into account the concerns and requirements imposed by the different stakeholders. To explore this new field, it is important to understand the different dimensions of operator models and how they are interrelated.

### Definition of an operator model

An operator model is a logical construct that connects different network dimensions and, thereby, defines a concrete instantiation of the relationship between every single pair of items belonging to two dimensions, either in the form of a connection between items or by pointing to another item of a third dimension. Therefore, a concrete operator model defines the following:

- A particular set of tasks during the entire lifecycle of a private 5G network,
- A particular set of stakeholders that are involved during the entire lifecycle of a private 5G network,
- Particular information about the stakeholders that are involved in (or responsible for) a certain task,
- A particular set of 5G-related elements as well as non-5G-related elements, as well as information about the private 5G network lifecycle tasks in which they play a role, and
- The definition of which stakeholder owns (initially as well as during the lifecycle) and which stakeholder governs a certain element.

**Fig. 5:**
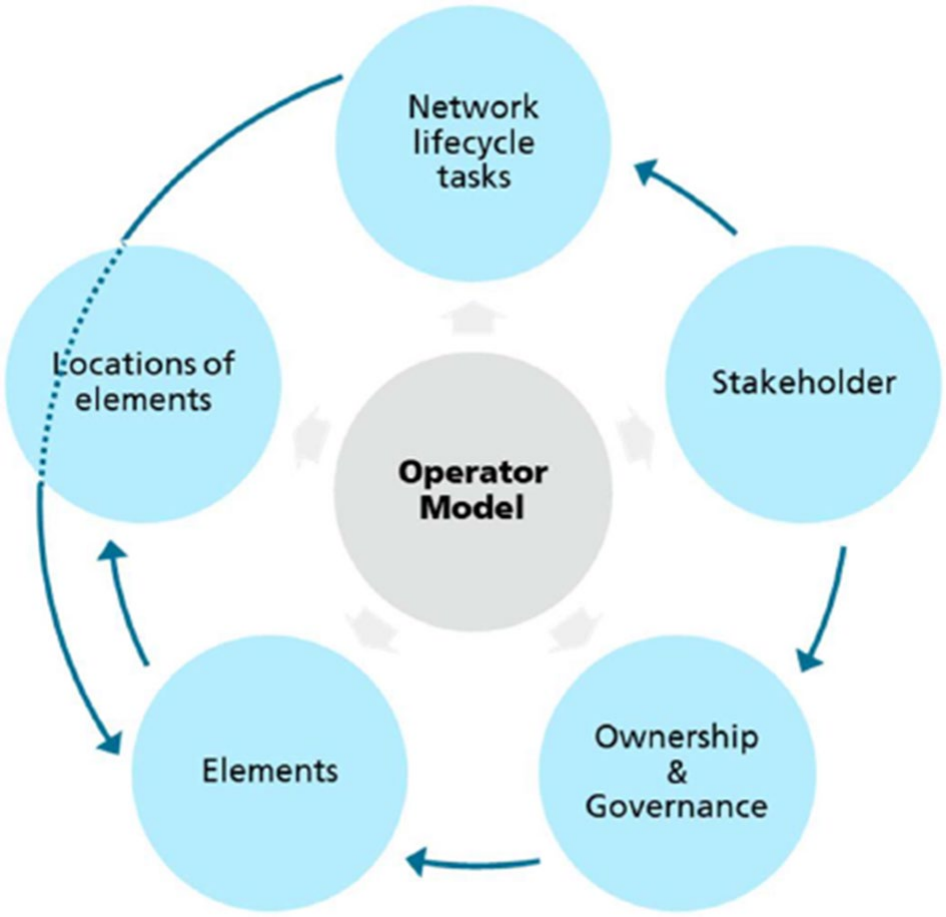
Interrelation between Operation Model Dimensions

Five different and interrelated dimensions can be derived and are depicted in Fig. 5.

**Table 3:** Description of stakeholders

Stakeholder	Description
Enterprise (E)	The E is the owner or manager of the premises and is responsible for the long-term innovation, efficiency and profitability of its operation. In large enterprises, teams can be dedicated to centrally or decentrally manage IT systems
Mobile Network Operator (MNO)	The MNO operates its mobile network infrastructure to provide connectivity to end-users. It merges the roles of mobile service provider and infrastructure provider
Network Equipment Vendor (NEV)	The NEVs or the hardware and software equipment manufacturers are responsible for building and delivering the hardware and software that compose the network infrastructure
Cloud Provider (CP)	The CP or the data network operator is a third-party company offering a cloud-based platform, infrastructure, application, or storage services
Service Provider (SP)	A SP is the entity that offers services to consumers. It can take the local operator role specialized for the specific facility. It can provide radio and core service, cloud services, management service, IoT services or security service. As discussed in [29, 30], this includes software network function providers, IT service providers, network service providers, communication service providers and online/over-the-top service providers
Third-party system integrator (3SI)	The 3SI is a third-party company, specialized in bringing together component subsystems into a whole and ensuring that those subsystems function together. The 3SI proposes a broad range of skills including software, system architecture and enterprise architecture, software and hardware engineering and interface protocols
Third-party network/ radio planner (3NP)	The 3NP is a third-party company, specialized in the process of proposing locations, configurations and settings of the new network nodes to be rolled out in the private 5G Network. Its main objectives are to implement an economically efficient network infrastructure, to obtain sufficient coverage over a target area and to provide the demanded network capacity by taking into account the specification of technology-dependent parameters
Brokers	They act as intermediaries between the different stakeholders. This could be the E, MNO, SP, 3SI or 3NP
Third-party WAN operator (3WO)	The 3WO is the owner, in whole or in part, of the WAN infrastructure, and makes its assets available as a service
Third-party Enterprise /Community (3EC)	The 3EC can participate regarding the private network, for example, external facility owner if an access point must be installed at a third party roof top
Government (G)	Government or office of communications / regulation is licensing spectrum or certifying products
Tenants	A tenant is a consumer of a virtual network service. It depends on the business model (i.e., business-to-business, business-to-consumers, business-to-government). A tenant renting a slice will typically specify which users can utilize that slice
Users	The users are typically called subscribers of mobile connectivity service. In a factory environment, such as a shop floor, the user of the technology is usually the factory personnel (e.g., machine builders, machine operators, local manufacturing IT management personnel, logistics workers)

**Elements** include 5G and non-5G system components, as well as all other physical and non-physical materials, information, etc., that are involved during the lifecycle of a private 5G network. Elements must be clearly known in the operator model in order to understand how they relate to their locations and stakeholders in terms of governance and ownership. In a private 5G network, **ownership** relates to the stakeholder that manufactures, produces and owns elements, and **governance** defines the stakeholder responsible for managing and operating a certain element. **Stakeholders** are organizations, institutions, persons, etc., involved during the entire lifecycle of the network. Stakeholder responsibilities are defined with respect to all network lifecycle tasks, including deployment of elements, operations and maintenance, etc. There is often more than one stakeholder involved in building and operating the private 5G network. Possible **locations of elements** include enterprise data center, enterprise headquarters data center, enterprise site, service/cloud provider central cloud, MNO central cloud, MNO edge cloud, and MNO site. The locations of elements specify the distribution of 5G elements, i.e., the 5G deployment model or architecture, which has some implications with respect to the operator model, operation and management. **Tasks during the lifecycle** points out that the private 5G network must clearly know the detail of its lifecycle subtask. This dimension can be used to know which tasks are involved in building the private 5G network.

### Description of the different dimensions

#### Ownership and governance

As discussed in [31], the sense of network ownership has evolved. Traditionally, the operator has owned the physical communication links, service infrastructure and customer relationships. This model has been increasingly challenged and transformed by virtual network operators, infrastructure sharing, the current trend of asset divestiture, and specialized infrastructure operators. Effectively, most end-to-end connections will pass through a multitude of stakeholders, who will not be bound by static service level agreements, but will need to pass through a rich ecosystem of dynamic technical (and economic) relationships. Ownership and governance of individual elements, especially network elements, are crucial aspects of operator models. If one stakeholder owns a network element, restrictions on governance and access to that network element by another stakeholder can be expected. In this regard, the following definitions are important: **Initial ownership** is the designer or developer of elements that will be used in the private 5G Network. **Owning Stakeholder** is the legal proprietor of the deployed element (e.g., physical infrastructure, licenses). **Governing Stakeholder** is the stakeholder responsible for the management and operation of the element in question. Management and operation tasks during the lifecycle of the private 5G Network can also be delegated by the governing stakeholder to another stakeholder, such as a subcontractor, while remaining responsible and liable.

**Table 4:** Description of 5G element

5G element	Description
Unified Data Management (Core-UDM)	The Core-UDM manages the subscriber information that is used for admission control and for defining the data path policies. Furthermore, it manages root keys for confidentiality and integrity protection of the data and control planes
Authentication Server Function (Core-AUSF)	The Core-AUSF is responsible to authenticate the users Session Management Function (Core-SMF) The Session Management Function (SMF) is responsible for the data path setup and tracking and terminating based on the policy function
Access and Mobility Management Function (Core-AMF)	The Core-AMF implements the access control and mobility aspects of the user context
User Plane Function (Core-UPF)	The Core-UPF defines the data path characteristics based on the users requirements and policy
Network Exposure Function (Core-NEF)	The Core-NEF provides a means to securely expose the services and capabilities provided by 3GPP network functions
Transport Network (TN)	The TN that is used to carry traffic between the 5G RAN and 5G Core network
Radio Access Network—Distributed Unit (RAN-DU)	The RAN-DU is responsible for real time L1 and L2 scheduling functions. RAN-DU sits close to the radio unit and runs the RLC, MAC, and parts of the PHY layer. This logical node includes a subset of the eNB/gNB functions, depending on the functional split option, and its operation is controlled by the RAN-CU
Radio Access Network—Central Unit (RAN-CU)	The RAN-CU is responsible for non-real time, higher L2 and L3. RAN-CU runs the RRC and PDCP layers. The split architecture enables a 5G network to utilize different distribution of protocol stacks between RAN-CU and RAN-DUs depending on midhaul availability and network design. It is a logical node that includes the gNB functions like transfer of user data, mobility control, RAN sharing, positioning, session management etc., with the exception of functions that are allocated exclusively to the RAN-DU. The RAN-CU controls the operation of several RAN-DUs over the midhaul interface
Subscriber Identity Module (SIM)	The SIM is a fundamental element of the cellular system, because it allows authenticating the validity of a terminal as it tries to access the network. It contains the unique identifier of the subscriber and the related security keys
5G Operation, Administration and Management (5G OAM)	5G OAM systems, such as the operation support system and the business support system, are complex applications that are required for a proper network configuration, operation and management, and for billing of customers (subscribers)
Spectrum	The electromagnetic spectrum is, for most parts, not a free resource, but in fact allocated and regulated into frequency bands by government bodies. Some of these frequency bands are unlicensed, which means that anyone who wants to use the spectrum can do so. Most of the spectrum however is licensed, which means that the license holder is the only authorized user of that spectrum range
Control Plane Data	Control plane is concerned with protocols, which control the radio access bearers and the connection between the UE and the network

#### Stakeholders involved in operator models

During the lifecycle of a private 5G network, there are important stakeholders involved who are responsible for carrying out specific tasks on network elements and other components. As discussed in [29, 30, 32], some of these stakeholders are also potential owners and governing parties. Table 3 collects all important stakeholders for the analysis and evaluation of the operator model dimensions and explicit operator models.

#### Elements and aspects relevant for ownership and governance

The operator models address the roles and responsibilities for certain tasks on a large number of 5G-related and non-5G elements during the private 5G network lifecycle, which are collected thereafter.

The elements that are relevant to ownership and governance, as well as for the lifecycle of the private 5G network and that are directly related to the 5G system, are described in Table 4. All these elements can be attributed to the 5G CN, 5G RAN, the User Equipment (UE) or the 5G operations and management system. The provided list is not exhaustive but presents the most important elements in this context.

In addition to 5G components, further non-5G elements are relevant for ownership and governance and, most importantly, for access and control by a number of stakeholders. These elements, which play a crucial role during the private 5G lifecycle, are collected and described in Table 5.

**Table 5:** Description of non-5G element

Non-5G element	Description
Application	There exists a plethora of different applications, which can be offloaded to a MEC platform. In the industrial domain, such applications range from simple data collection and database systems to control logic functions of controllers to more complex systems, such as manufacturing execution systems or even enterprise resource planning software. Depending on the type of the application, the MEC platform is either deeply integrated with the 5G System and located close to a machine or production line, or it provides computing capabilities for a large number of machines, sensors etc., that can even span across multiple factories
MEC Platform	The purpose of the edge-computing platform is to carry applications and connect telecom operators’ network equipment, and thus telecom operators usually own the edge-computing platform. Owners of the edge-computing platform must maintain the network connectivity and assist in generating applications of the platform
User Plane Data	User plane is responsible for the transfer of user data, such as voice or application data through the access stratum
Wide Area Network (WAN) Infrastructure	A WAN is a telecommunications network that extends over a large geographic area for the primary purpose of computer networking. WAN infrastructure may be privately owned or leased as a service from a third-party service provider, such as a telecommunications carrier, internet service provider, private IP network operator or cable company. For operator models, in which multiple stakeholders are involved carrying out OAM tasks remotely, the WAN infrastructure plays a significant role, e.g., regarding availability of the entire distributed system
Shop Floor	A shop floor is the area of a factory, machine shop, etc., where people work on machines, or the space in a retail establishment where goods are sold to consumers
Shop Floor Plan	The map of the factory including information about physical objects, such as machines, walls, production lines, etc.
Enterprise Network IT	An enterprise IT network is the backbone for facilitating an organization’s communications and consists of physical and virtual networks and protocols that serve the dual purpose of connecting all users, computers and devices throughout departments on a local area network to applications in the data center and cloud as well as facilitating access to network data and analytics. These information networks can include local area networks, WANs, intranets and extranets. The enterprise network IT plays an important role regarding the deployment and integration of a private 5G network, especially with respect to IT security
Third-party Cloud Platform	It is a third-party company platform proposing the delivery of computing services—including servers, storage, databases, networking, software, analytics, and intelligence—over the Internet to offer faster innovation, flexible resources, and economies of scale
Enterprise operations and maintenance (OAM) Systems	Enterprise OAM systems plans and executes activities such as operating the system, or monitoring system performance. Such systems become important, when existing network infrastructures converge with the private 5G infrastructure
Enterprise Personnel and/or End Device Database	It corresponds to the database of enterprise personnel to provide them access or to end device such as computers, robots, machines, cameras, etc.
Power Supply	The power supply is a hardware component or network that supplies power to electrical devices. The plan of the enterprise power grid will also be necessary to deploy powered devices of private 5G Networks

#### Location

Elements, and those related to 5G in particular, can be placed in a number of different locations, which has a number of implications with respect to the ability of stakeholders to operate and manage the system, IT security as data must be sent over geographically distributed networks. In light of private 5G networks, a number of different locations need to be considered.

- Enterprise site is the physical location including the infrastructure on the enterprise premises, where 5G end devices are installed, either inside the factory or a plant.
- Enterprise datacenter is an enterprise-owned and managed data center infrastructure that is logically or physically separated from the IT infrastructure on the enterprise site, meaning that it can potentially be located offsite. An enterprise may have multiple such enterprise data centers and more than one enterprise site may be connected to the enterprise data center.
- Enterprise headquarter datacenter is a datacenter infrastructure owned and governed by the enterprise.
- MNO site is the area where the MNO builds the base stations. This area may be equivalent to the enterprise site in the case of a dedicated indoor deployment inside a factory, but it may also be a separate location, for example, if the private network shares the MNO outdoor RAN.
- MNO edge cloud is a small-localized datacenter infrastructure owned and governed by an MNO.
- MNO central cloud is a (partially) public cloud infrastructure owned and governed by an MNO.
- Service/cloud provider central cloud is a (partially) public cloud infrastructure owned and governed by a third-party service/cloud provider.

**Fig. 6:**
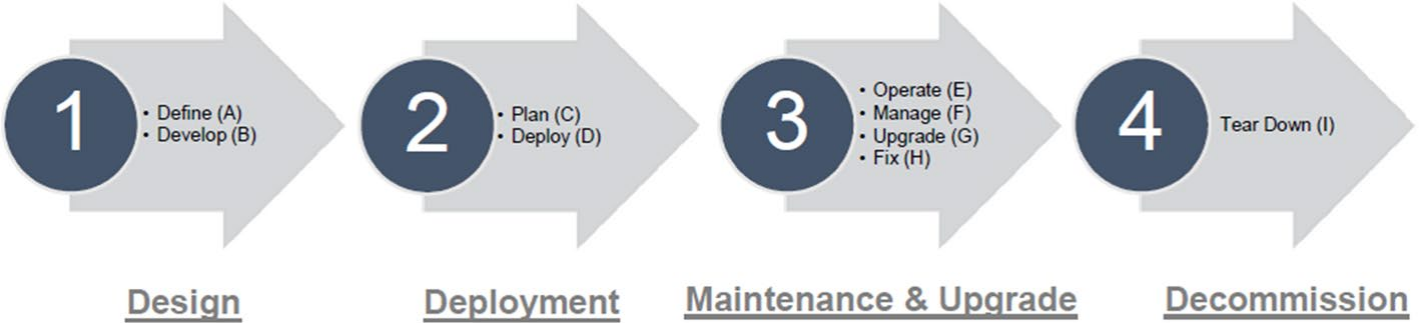
Lifecycle of Private 5G Networks

#### Private 5G network lifecycle

As operator models are concerned with assigning roles and responsibilities to stakeholders for all the relevant tasks, from defining aspects of the 5G network to network deployment and eventually decommissioning, it is important to detail the lifecycle of a private 5G network and the associated tasks. In general, a lifecycle model helps ensure that delivered private 5G networks meet stakeholder requirements, particularly those of the enterprise, provides strong management controls over projects, and makes the management process efficient. The lifecycle of a private 5G networks can be composed of four phases and nine high-level tasks, as depicted in Fig. 6

The first phase involves the design of the private 5G network, where the objectives are to determine the business goals, define a high-level design and develop all the elements of the private 5G network solution. The second phase includes the deployment of the solution at the selected site, such as the factory floor. The objectives are to plan (infrastructure and radio) and adapt the solution to the specific scenario of the particular site, and to configure, integrate, deploy and test the complete solution. In the third phase, the main concerns are the operation, maintenance and network updates, e.g., hardware and software. The objectives are to operate the network through daily management and to optimize the network through proactive management and design improvements. Finally, the last phase includes the decommissioning of the network.

### Dimensions of 5G network ownership and governance

#### SIM

In 5G systems the SIM card is capable of supporting seamless global roaming using roaming procedure steering, which can deal with parameters such as operator controlled Public Land Mobile Networks (PLMNs) to provide roaming service. An embedded-SIM (eSIM) or embedded universal integrated circuit card as a programmable SIM card can be directly embedded into a device. The eSIM allows consumers to simultaneously store multiple operator profiles on a device, and switch between them remotely, although only one can be used at a time. In the context of private 5G networks, SIM cards are typically issued to each user equipment by the stakeholder involved in the management of the core network.

#### RAN

The Radio Access Network (RAN) is in many ways the most important asset of a mobile system as its deployment and interconnection are subject not only to coverage and KPI requirements but also to a number of constraints imposed by the regulator, the real estate market and the telecom market. In fact, radio coverage design must take into account the signal-to-interference-and-noise ratio caused by incumbent RAN deployments, the maximum radiated power allowed in that region, the availability of sites to install equipment and the resulting capital and operational expenditure. In order to reduce costs, RAN sharing models have been explored, allowing different PLMNs to be supported by the same RAN system. National roaming and MVNO enable a communication service provider to deliver service in a region even if it does not have a RAN system there. Dedicated Core networks (DECOR) and network slicing technologies enable mobile communication service to be provided to private subjects when they do not have a mobile system at all. Another model of RAN sharing is the so-called neutral host, in which the RAN infrastructure is not owned by any of the MNOs whose PLMNs are supported by that infrastructure, and the infrastructure owner also announces its own private PLMN. This is an architecture defined by the MulteFire alliance and citizen broadband radio service Alliances, and targets tower companies that may also be service providers. The stakeholders involved in the RAN ownership and governance are quite heterogeneous (MNO, MVNOs and roaming MNOs, equipment and service providers, tower companies, building management companies, national regulator or local administrations).

#### Core

Similar to the RAN, the Core Network (CN) is not necessarily owned by the stakeholder that defines which subscribers and devices are allow to use the 5G communication service. In fact, that organization can use the mobile network offered by a traditional MNO or an equipment/service provider as a service, so even subscriber management is delegated to a third party, technologies such as dedicated core network and network slicing enable this scenario. Nevertheless, such organization is still responsible for the administration and provisioning of the subscribers database, including their service profile, and for the distribution and configuration of physical SIM cards or eSIM to users. The Authentication Server Function (AUSF)/Unified Data Management (UDM) are then responsible for authenticating the device by exploiting the information contained in its SIM card, such as the international mobile subscriber identity, the subscription permanent identified and relative keys. In other cases, however, the main stakeholder may own the entire core network, or act as an MVNO, owning only a few core network functions. As a consequence, depending on the specific case, SIM cards may be issued by the private 5G network provider or by the MNO. Stakeholders involved in the CN ownership and governance are usually MNO, MVNOs and roaming MNOs, equipment and service providers or resellers and partners of the equipment and service providers.

#### MEC

The owners of the edge computing platform must maintain network connectivity and assist in generating applications from the platform. The method of generating applications is usually based on ETSI Network Function Virtualization (NFV) Management and Orchestration. Besides, they must also ensure that user packets are delivered to the correct applications and target terminals. The owners of the edge computing platform are telecom operators. Therefore, they must manage the operation and performance of the device on the platform and consider the overall security of the network transmission between devices. The edge computing platform must be considered as the appropriate amount of resources to generate devices to achieve maximum resource usage. Besides, it must also consider the traffic routing between devices. In data routing process, the telecom operator is responsible for the network routing of the edge computing platform away from the applications, sending data to the target applications, and processing the data completed by the applications to the target users. In addition to the typical scenarios mentioned above, non-network-related enterprises may also obtain the edge computing platform through buyouts or leases and then install the applications on the platform. In this case, the platform that the telecoms help build is inline with the requirements of enterprises, so it is a cooperative relationship. The enterprise is responsible for the cost of establishing the edge computing platform and the requirements to configure the edge computing platform. The telecom operator is in charge of the establishment of network connectivity, network transmission security, application onboarding functions, and transmission performance according to the requirements of enterprises.

#### Applications

There are a plethora of different applications that can be offloaded onto a MEC platform. In the industrial domain, these applications range from simple data collection and database systems to control logic functions of controllers to more complex systems, such as manufacturing execution systems or even enterprise resource planning software. Depending on the type of the application, the MEC platform is either deeply integrated into the 5G system and located close to a machine or production line, or it provides computing capabilities for a large number of machines, sensors, etc., that may even span multiple factories. Application ownership and governance is more flexible than that of the MEC platform, as applications can be provided by many suppliers, such as telecom operators, enterprises, or application providers, and each can manage their respective applications individually. In terms of application ownership, regardless of who owns these applications, they must all maintain basic network connectivity, data security, and resource utilization.

#### Transport network

Ownership, governance and management of the transport network, such as a Wide Area Network (WAN), can be critical aspects of operating private 5G networks. An enterprise backbone WAN may be owned and operated by the same enterprise, but it may also be owned and managed by one or more third parties, which are neither the enterprise nor the M(V)NO. There are two cases in which the backbone must be used to transfer signaling, operation and management data or even user data: (i) when there is no direct connection between the site’s IT infrastructure and the M(V)NO provider network, (ii) when the enterprise wants to implement more than one 5G access network across multiple geographically distributed sites, which are operated centrally by either the enterprise itself or by an M(V)NO.

**Fig. 7:**
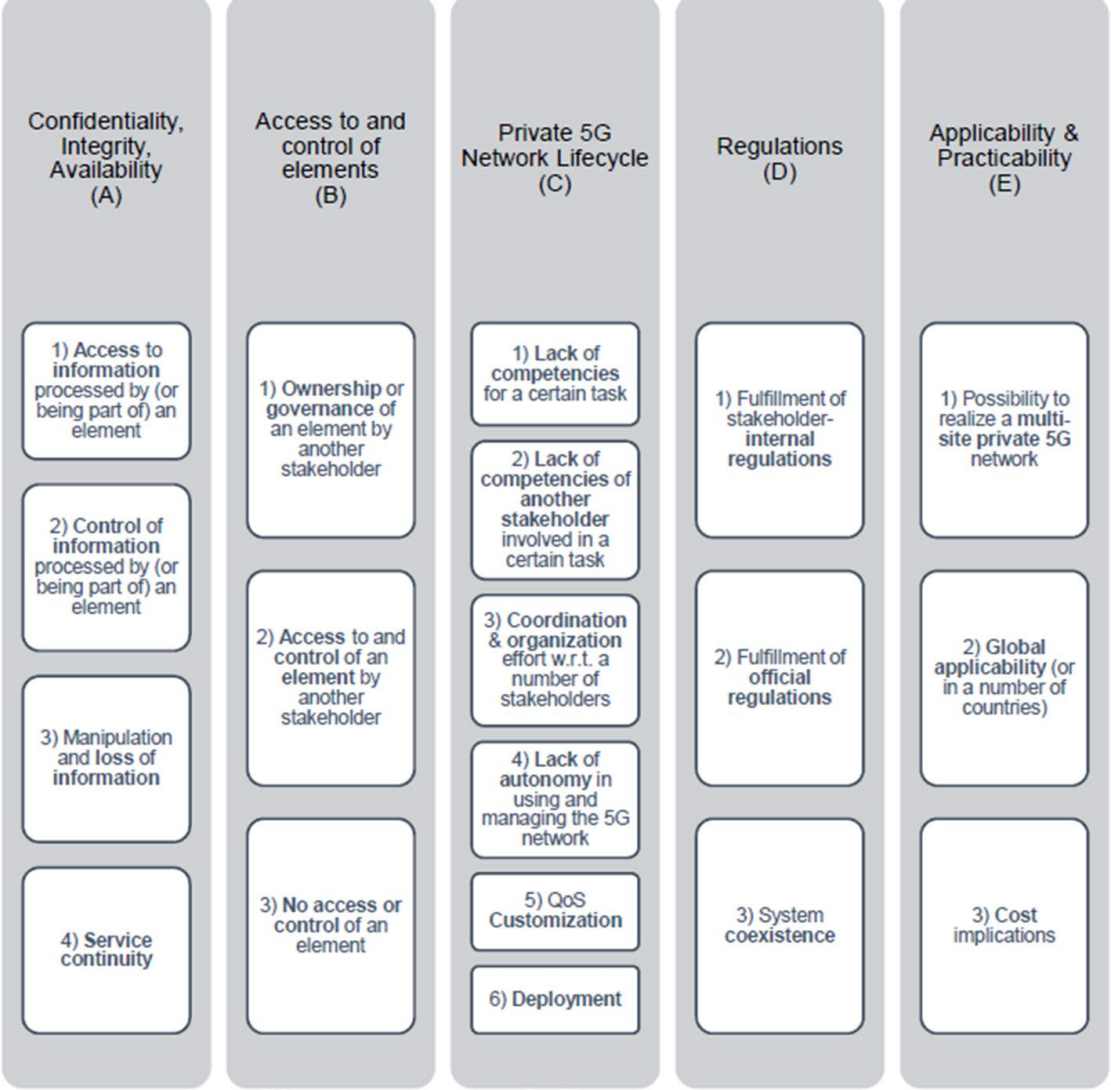
Categories for concerns regarding operator models

#### Operation, administration and management system

In the typical operator business, the operation support system and business support system are owned and used by the MNO to carry out the respective tasks. In light of the developments around private 5G networks and local, private spectrum, a number of other stakeholders may own and use the network management tools and, therefore, would also be responsible for all legal, technical and operational consequences. In particular, the owners may be any other service provider that is not an M(V)NO or even the enterprise, for which a private network is planned. In the latter case especially, the Operation, Administration and Management (OAM) system may be located and run in the enterprise data center or in the plant data center, so that OAM traffic remains essentially within the corporate network and can be easily protected by security mechanisms in accordance with enterprise-specific security regulations.

### Discussions about concerns and requirements of operator models

An operator model is defined by sets of stakeholders, tasks and elements, as well as information about which stakeholder is involved in a certain task and which stakeholder owns and governs the elements, in particular the 5G elements. In contrast, a deployment model (or architecture) specifies where certain elements are installed. Since the two aspects (operator and deployment models) go hand-in-hand, the location of 5G elements must also be explicitly considered when identifying concerns and requirements for operator models. Concerns may relate to each interface between dimensions, but also to the entire operator model construction. They may be related to the dimension itself, or to another aspect, such as an IT security concept, that is not explicitly part of an operator model.

In general, the concerns can be grouped into a number of categories such as confidentiality, integrity and availability; access to and control of elements; private 5G Network lifecycle; regulations; and applicability and practicability. Figure 7 shows them along with their subcategories, which themselves contain the actual concerns and requirements.

**Confidentiality, integrity and availability of information**  are the three main pillars in IT security, and are especially important for any enterprise using a private 5G network. Any information, such as production process data, must be properly protected, which is even more important if another stakeholder is involved in operating the underlying IT infrastructure. Due to its modularity, the 5G System allows for a flexible distribution of network functions, which are responsible for both the control plane and the data plane. The ability to access or even actually access the information processed by such an element can raise concerns for another stakeholder, in particular the enterprise. Other information that needs to be accessed and used during the design and deployment phases of the private 5G network lifecycle that is important to a stakeholder should be considered. Control of information and also control over how information is processed in terms of security measures are two other important aspects, which play a role in terms of concerns and requirements for operator models. Not only access to sensitive information by another party or an attacker but also its manipulation or loss can cause considerable damage to the data owner. Hence, stakeholders must also consider these concerns when designing an operator model. Availability refers not only to information but also to a service; in this case, the connectivity service of the private 5G network. These concerns are considered by the enterprise, the service provider and the MNO as well.

Because the deployment and operator models are intertwined, another group of concerns and requirements are related to the aspects of **access to and control of elements**, whether they are 5G elements or not. In contrast to information access and availability, this group is specifically related to the interactions (and their restrictions) of stakeholders involved in certain lifecycle tasks with the corresponding elements. Such concerns are then usually raised because other stakeholders are involved in certain tasks or by the fact that access and control of the elements is limited.

Private 5G networks offer new business opportunities and allow multiple stakeholders to coexist on their infrastructure to be tailored to specific business needs. It is not always easy to manage the **private 5G network lifecycle** and even less so when the requirements are very specific. Operator models need to assign roles and responsibilities to stakeholders in each task based on their competencies, give them enough autonomy to accomplish their action, organize each task and coordinate each stakeholder. The flexibility of the private 5G architecture enables customized network deployment and the support of heterogeneous use cases with different requirements. This flexibility makes network deployment more challenging. For the QoS customization, each stakeholder needs to exchange specific requests and requirements and some configuration options need to be available. Some concerns are related to the availability of non-5G elements to realize a deployment. If the deployment is limited by power, space, cooling or transport connection, enterprises must provide existing infrastructure or deploy new infrastructure for power supply, cooling or local area network infrastructure.

**Regulation** needs to take a harmonized approach that facilitates support for private 5G network deployment by all stakeholders (stakeholder-internally, officially). Internally, different enterprises may have different security requirements for private data or resources. These enterprises can discuss how to find a compromise between their respective security requirements to decide the most feasible security solutions. Regarding the spectrum regulation, the government manages the spectrum and provides enterprises with spectrum leasing services. Therefore, companies in different countries/regions may use different spectrum to work and must follow their regulations. In order to comply with official regulations, an enterprise may have concerns about the proper handling of the spectrum, especially in terms of properly managing interference with adjacent (private) networks.

**Fig. 8:**
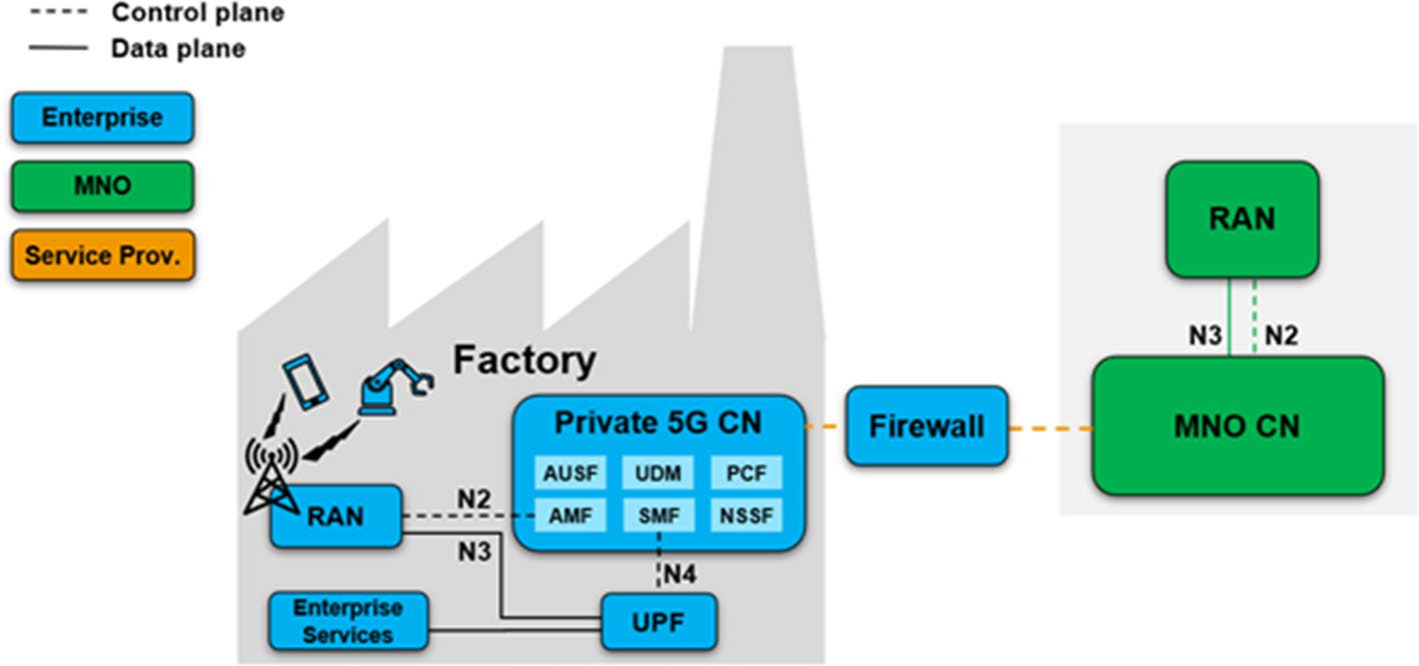
Fully private model. The private CN may optionally connect to a public MNO’s CN, as the NPN operator can conclude roaming agreements with one or more public network operators

For enterprises with multiple sites distributed across several countries, **global applicability and practicability** of the operator model is an important group of concerns. 5G offers a flexible, modular and programmable system architecture that enables a wide range of deployment scenarios. Nevertheless, multi-site deployments require interoperability, interconnection or shared models/components to mutualize costs, sometimes under different regulations, laws and architectures. The enterprise with multiple sites may be concerned about the added complexity of managing a multitude of different operator models, which can be a burden for monitoring and managing networks across sites. Operator models will be influenced by the cost involved with each specificity, performance requirement, coverage extension or additional support.

## Novel network architectures

When moving from traditional PLMNs to private 5G network deployments models, ownership and governance of the various network dimensions are shared across multiple stakeholders, as opposed to a single MNO. These dimensions, described in Sect. 4, cover a wide space of possible architecture and deployment options, as well as the different stakeholders involved. The choice of an architecture will depend on the specific functional and organizational requirements of the enterprises. This section presents four architecture options that are suitable for private network deployments. For each option, the stakeholders involved in all dimensions are (i) MNO/MVNO, i.e., the mobile network specific service provider, (ii) the enterprise, which includes the premises owner, the enterprise IT management team and the end users and (iii) the service provider, i.e., any party other than the M(V)NO or the enterprise. Depending on the actual realizations of the proposed architectural models, the stakeholders may be either **owning stakeholders** or **governing stakeholders**, as defined in Sect. 4.

Moreover, the possible deployment locations for hardware and software components are (in increasing order of distance from the network edge) the **edge cloud**, i.e., infrastructure located on or near the enterprise’s premises, the **enterprise’s datacenter/cloud**, i.e., a datacenter infrastructure owned and governed by the enterprise, possibly located off-site and the **central cloud**, i.e., a (partially) public cloud infrastructure owned and governed by an MNO and/or third-party service provider.

Each of the options presented in this section is representative of a broader class of architectures, as many adaptations and variations can be designed to meet specific real-world deployments. In general, however, private networks can be divided into two categories: private networks that are deployed as isolated and standalone networks and private networks that are deployed in conjunction with the public network operated by an MNO. The first category corresponds to the network configuration described in Sect. 5.1. The second category includes the three options described in Sects. 5.2, 5.3, and 5.4. These options offer a combination of public and private networks, and differ in the way this interaction works.

### Fully private infrastructure

When it comes to implementing Industry 4.0 and dedicated services for enterprises, the ability to have a highly available mobile network that also operates in isolation from the rest of the national network and can prioritize voice, video, data and IoT services is a key requirement. The fully private 5G model is the most appropriate solution in this context, as it preserves the privacy of data generated and consumed within the enterprise. The solution also integrates the intranet and cloud services that are specific to the enterprise itself.

As shown in Fig. 8, a fully private ownership provides for the enterprise to own almost all dimensions, i.e., spectrum, RAN, MEC, CN, and applications. The only dimensions excluded are the OAM system and the transport network, which may be owned by a service provider or operator. Typically, for large enterprises, this model allows a dedicated enterprise IT management staff to manage the private network. In the case of an enterprise deployment, the CN is integrated and the enterprise network, along with the enterprise IT management team, is responsible for assigning the appropriate Internet Protocol (IP) addresses to the mobile devices. This setup allows the IT team to apply the same policy (firewall, network address translation, traffic separation, etc.) for both fixed and mobile users in the enterprise.

With all dimensions of ownership under the control of the enterprise, the fully private infrastructure model offers the highest degree of flexibility to tailor the 5G system to specific enterprise requirements. Particularly with respect to enterprise-specific security regulations, this model is likely to meet the associated requirements, since all data carried by the 5G system, including user, control and OAM traffic, is fully controlled by the enterprise. If one chooses to design the system accordingly, no traffic leaves the corporate IT network, thus exposing no potential vulnerabilities to external malicious actors. However, among the deployment models discussed in this section, this one places the greatest burden on the enterprise, which assumes most of the responsibilities during all phases of the deployment. Specific knowledge is required for 5G system planning and operation, and it is likely that this knowledge does not exist within an enterprise. Of course, the enterprise may choose to outsource the planning and/or operation of either dimension of ownership to an external service provider.

**Fig. 9:**
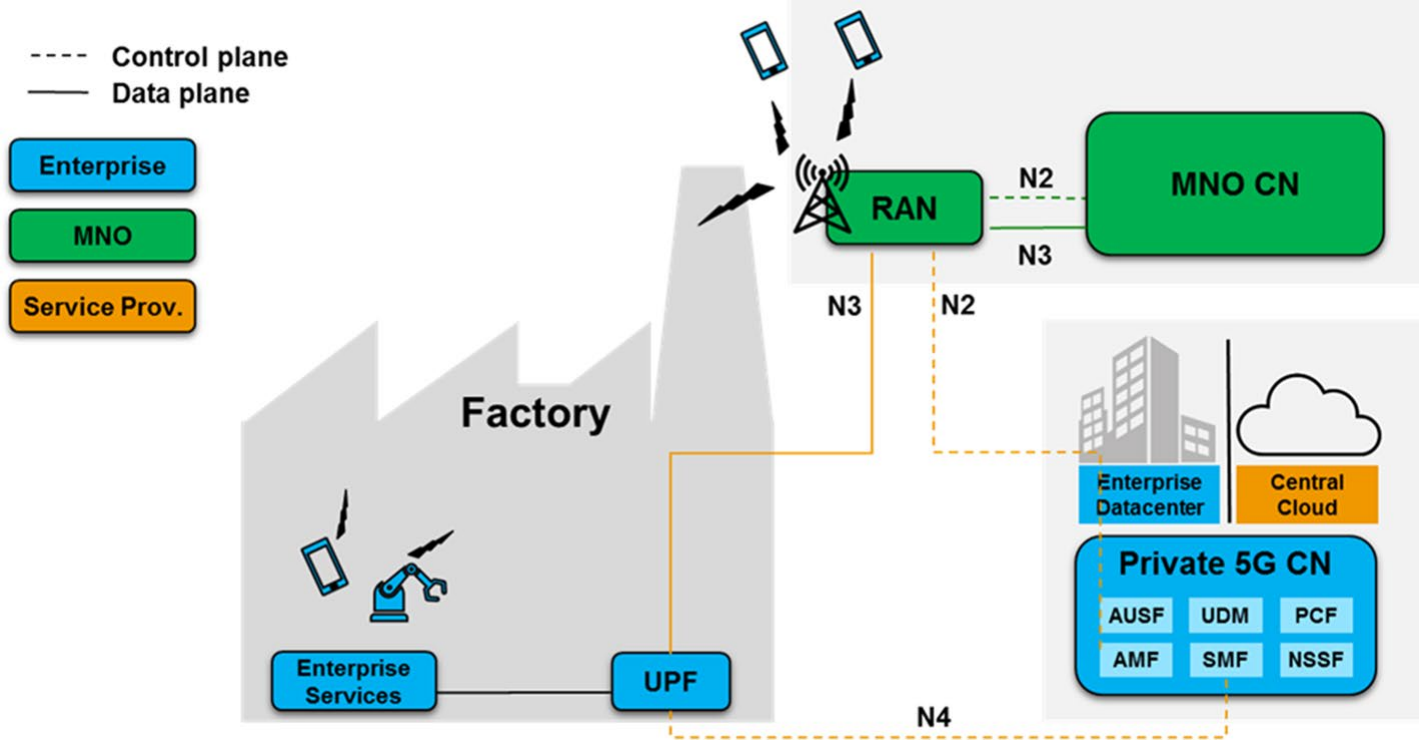
MVNO model

### MVNO model

In this scenario, the private and public network share part of the RAN, while the other network elements remain separate. All user plane data related to the private network is terminated on the premises. The logical architecture is shown in Fig. 9.

The private 5G network includes the RAN, Core, OAM system, transport network, MEC platform, applications, spectrum and SIM. The MVNO model calls for the enterprise to own almost all dimensions except the RAN and transport network. The enterprise deploys its own core network, MEC platform and applications, while the RAN is shared and connected to both the MNO and the private CN. The radio network is accessible to both public and private users. Depending on the scale of the enterprise, the OAM system and transport network may be owned by a service provider or an MNO. The same is true for the governing stakeholders, where each network dimension or element can be managed by the enterprise itself, the service provider or an operator.

Since the enterprise deploys its own private CN, the degree of compliance is high in terms of subscriber management. Furthermore, third party application programmer interfaces should be available in this shared RAN architecture, allowing the enterprise to have full access to operation and management functions. It can monitor the network status to troubleshoot faults or even identify potential problems as early as possible.

In this model, the RAN is shared and connected to both MNO and private core network. This requires the governing stakeholder of RAN to consider the QoS requirements of both networks. To this end, the enterprise has to reach the RAN sharing agreement with the MNO to ensure that the enterprise’s service requirements are met end-to-end. This can be achieved by using efficient radio resource allocation mechanisms.

In the fully private model described in Sect. 5.1, the enterprise purchases, owns and manages the private 5G network. Instead, the MVNO model is better suited for enterprises that want to outsource the day-to-day operations of the RAN which requires spectrum availability and technical expertise to optimize hundreds of parameters in the radio network. In addition, the user plane data will remain on the enterprise premises through the self-managed core network.

**Fig. 10:**
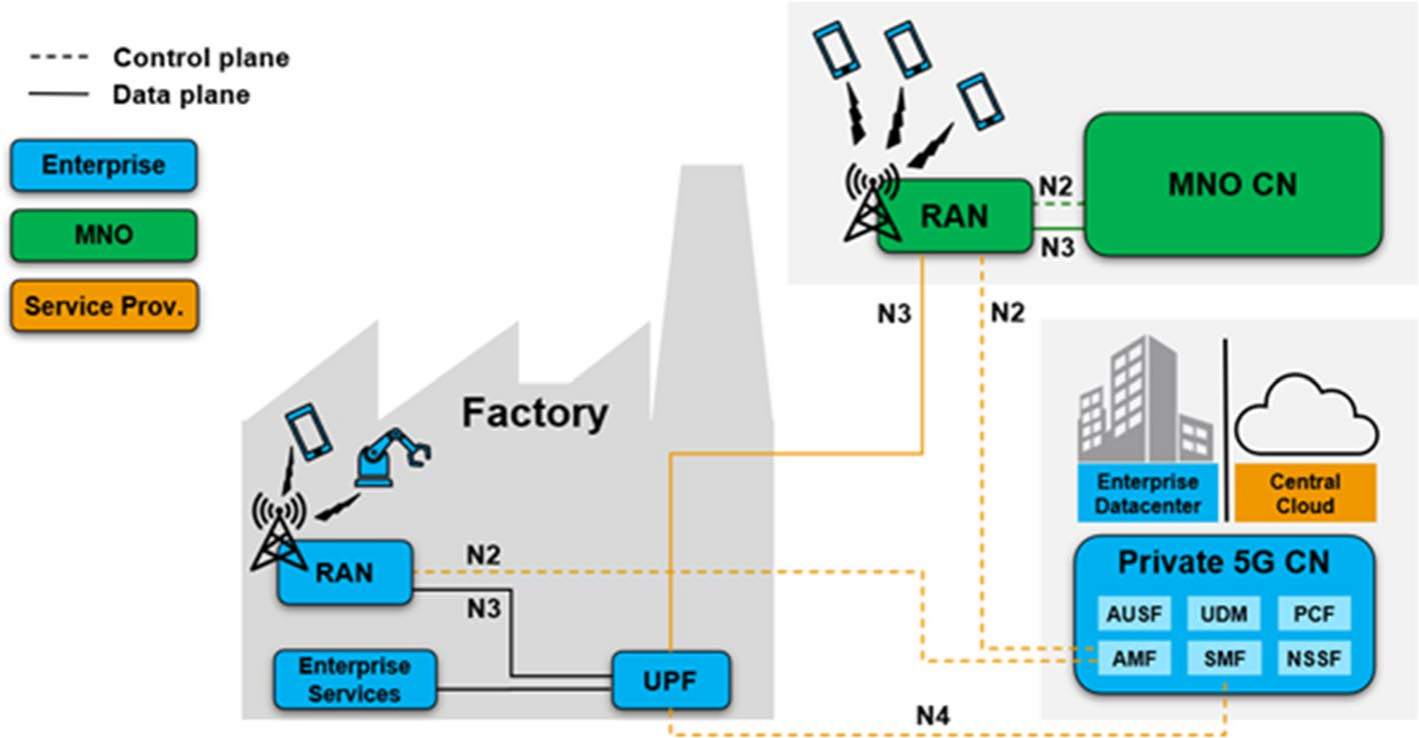
Hybrid model. UEs can connect to the private CN by accessing from a private RAN or a public one. The enterprise’s CN may be placed in a private datacenter or a central public cloud

### Hybrid model

The hybrid model can be viewed as a combination of the fully private and MVNO models. As shown in Fig. 10, the enterprise hosts a local private RAN and MEC platform, which are connected to a private CN, also owned by the enterprise. However, radio access of enterprise’s UEs can also be roamed via the public MNO RAN, which forwards control and management traffic to the private CN. In the hybrid model, the CN may be split into a centralized control center (typically containing the 5G control-plane elements), which interacts with the local RAN and devices through locally deployed edge nodes (containing User-Plane Function (UPF) and the MEC platform), as shown in Fig. 10. This platform allows the deployment and management of multiple distributed private networks, each anchored by an edge node. The edge node resides inside the enterprise firewall and keeps traffic and user data local to meet low latency, data security and edge computing requirements. The edge node can have a very small footprint to be deployed in each commercial building, factory, warehouse or enterprise, or it can be deployed at an aggregation point, to be used by multiple private networks.

The hybrid model can be viewed as part of a long-term transition strategy: the enterprise can start outsourcing with a simple MVNO model (see Sect. 5.2), in case an adequate enterprise IT management team is not put in place initially. Once a favorable status is reached, the enterprise can begin a transition to a fully private network (see Sect. 5.1), in which the entire network is owned by the enterprise.

If SIM cards are allowed to roam between the private and public RAN, the manner in which they are ordered and deployed must be agreed upon between the enterprise and the MNO. Furthermore, SIM authentication must be managed to enable seamless access between the different owned RANs. Moreover, when configuring private communication, the governing stakeholder can grant access to a specific group of UE SIMs between private and public RANs to maintain group isolation. The power of the hybrid model lies in the centralized management system that serves as a single point of integration and control for the distributed edge nodes. This allows the governing stakeholder to take an active role in monitoring the connectivity status of the edge nodes and devices, analyzing outage and performance, and managing services. The control center allows the Communication Service Provider (CSP), resellers and end-customers to deploy, manage, monitor and control the entire network (in the case of the CSP) or their relevant network modules (for system integrators and tenants). CSPs can also allocate SIMs through a waterfall procedure to resellers who can then further distribute and activate the SIMs to end tenants.

This approach eliminates the cost and complexity associated with traditional Evolved Packet Core (EPC) deployments and enables low-touch, low-cost deployments with full local offload of client traffic and data. It allows low-latency applications and compliance with traffic and data privacy policies and requirements.

**Fig. 11:**
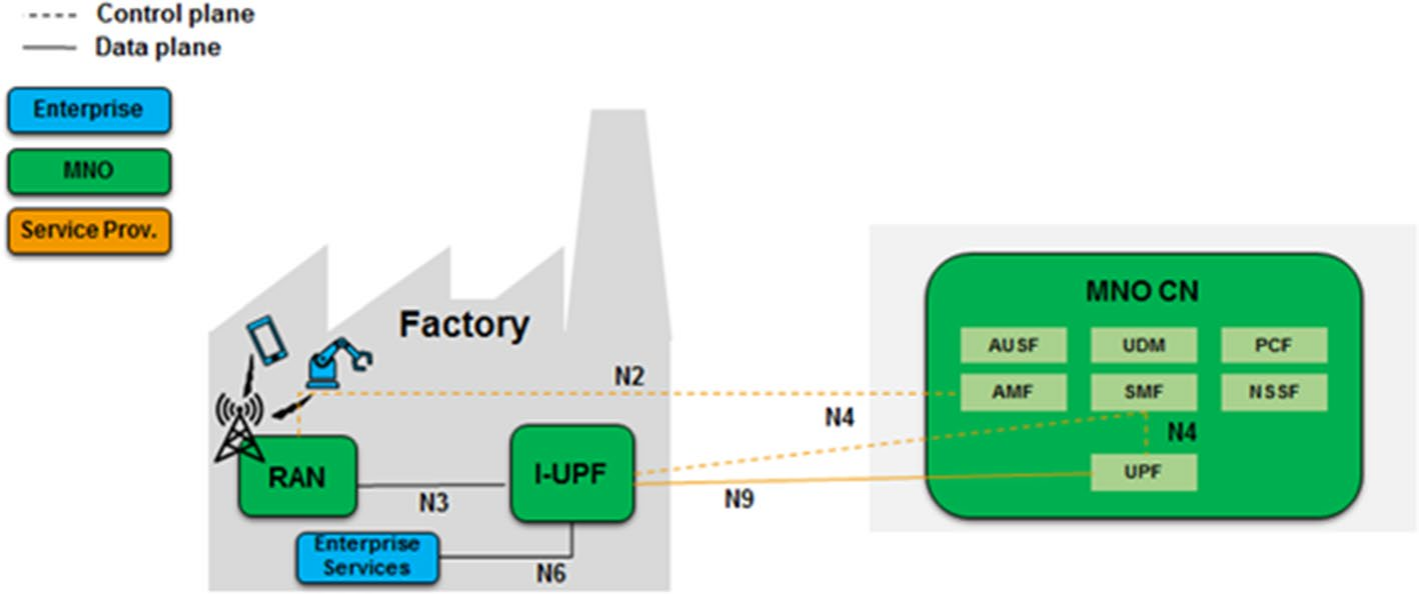
MNO’s Private Core Network architecture with I-UPF local breakout

**Fig. 12:**
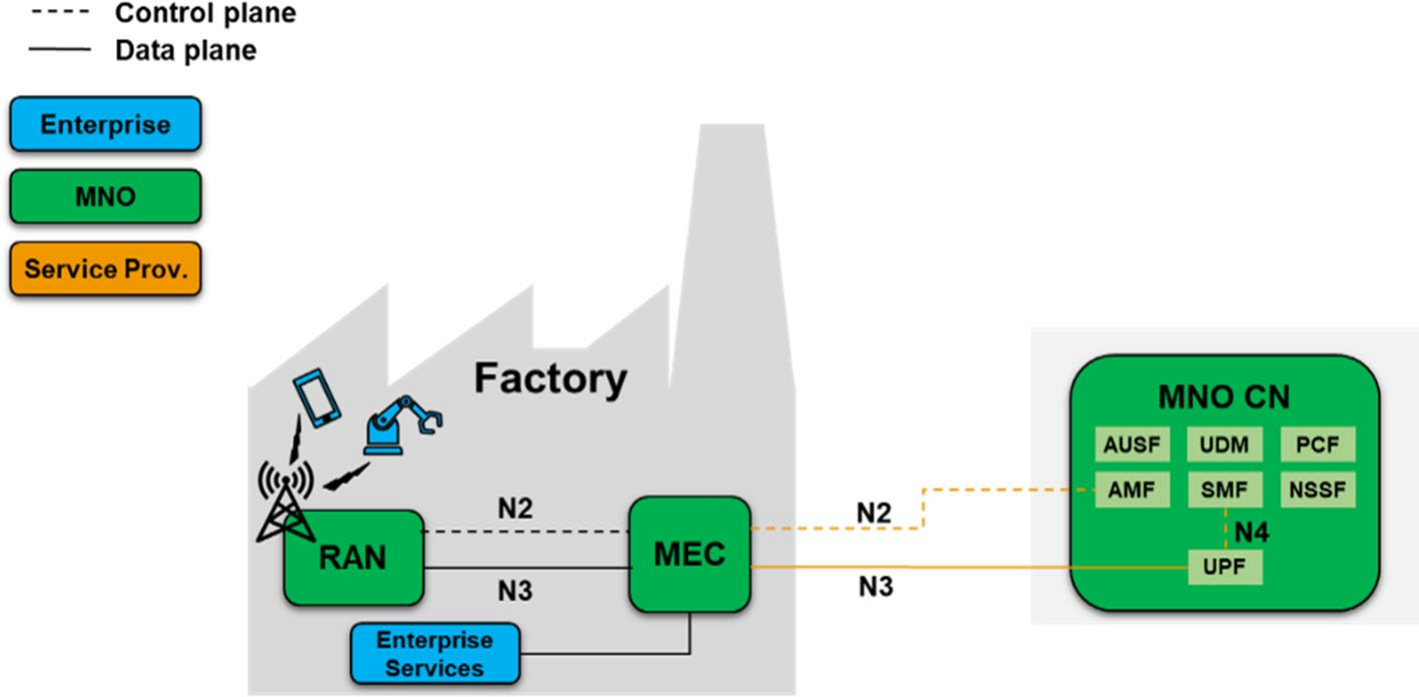
MNO’s Private Core Network architecture bump-in-the-wire edge breakout option

### MNO’s private core network

The MNO private network architectures is similar to the MVNO model, but the CN, transport network, spectrum, and SIM cards used by the enterprise are owned by the operator. This model is compatible with end-to-end network slicing technology, so that CN and RAN resources can be separated between different enterprises. We consider two sub-cases of this architecture: the Multi-Operator Core Network (MOCN) architecture and the DECOR architecture.

For the MOCN architecture, the same RAN is shared at a site, so operators can share the same RAN and spectrum resources to reduce hardware cost. The UE, RAN and Access and Mobility management Function (AMF) should allow operators to use more than one PLMN ID. The 5G MOCN also can support the New Generation RAN (NG-RAN) sharing with or without multiple cell identity broadcast. For the DECOR architecture, operators can deploy more than one core network in a single PLMN for different subscriber and UE types. Based on the “UE Usage Type” declared by a UE, the CN can identify which type of UE belongs to which DECOR, and provide the isolated slice resource to serve the specific type of end devices.

5G systems compliant with the 3GPP R16 standard allow for Intermediate-UPFs (I-UPFs) in the 5G CN architecture. The I-UPFs between the packet data unit session anchor UPF and the NG-RAN can be used to support the local data flow, which uses the N3 tunnel connecting to the NG-RAN node and via the N6 interface connecting to the utility at the edge or local site, as depicted in Fig. 11. In this architecture, the enterprise can have its own data flow transport without backhauling to the operator’s data center to achieve greater efficiency and lower communication latency.

Instead, the bump-in-the-wire mode consists of a dedicated RAN, on-premise MEC, and an operator-built CN, as shown in Fig. 12. The USIM cards also belong to the MNO. It is convenient to use the same USIM card between private and public networks. Enterprise applications are deployed on the on-premise MEC server. Since the RAN is connected to the MNO’s CN, operators help enterprises deploy the MEC and connect to their internal applications. This architecture distinguishes between internal and external areas of the enterprise through dedicated base stations.

In the MNO’s private core network model, the operator plays a central role, as it provides most of the network components. Enterprises essentially only have to prepare their own applications and service requirements requested by the use cases in the enterprise’s intranet. For this division of responsibilities, the enterprise and operators may need to discuss the information sharing mechanism between the enterprise and operators for the network OAM system. Similarly, the enterprise and operators should ensure that they clarify the authority of monitoring systems and provide fault management functions, and then discuss the specifications that the operators plan to implement in the enterprises to support those services. In addition, in the MOCN and the DECOR architecture options, the end user has no control over the network, only the management of SIM cards and IP address assignment.

### Discussions about private network use cases and architecture

Different use case scenarios for private 5G inter-site deployments, i.e., having multiple infrastructures interconnected or integrated at different physical or geographic locations, can be described in this paper. These scenarios can be grouped into two categories: intra-enterprise and inter-enterprise scenarios. In the intra-enterprise scenarios, user plane data are produced and consumed within a single enterprise, which may nevertheless have different geographical locations far apart, such as office buildings and production plants. To design a private mobile network to meet such scenarios, three general architectural choices can be considered for the CN and its functions. In the first framework, these functions can be distributed among the different sites of the enterprise, for example for reasons of increased performance or reliability. However, this choice is made at the cost of a more complex management. A second option is to centralize all CN functions. This has the advantage of reducing the complexity and effort of managing and orchestrating the network at the different sites. Finally, a third intermediate stage is possible, called the hybrid model. In this model, a central CN is accompanied by replication of some critical control plane functions to specific distributed sites, such as a production plant, to enable services such as ultra-reliable low-latency communications. These network functions may include the application function, which, for example, must interact with the 5G Core and manufacturing applications; or, for privacy reasons, the user plane function in its role as a gateway to a central database. In contrast to intra-enterprise scenarios, inter-enterprise scenarios include business cases where user plane data are shared between different enterprises. In such a setting, communication protocols and network architectural choices must obey even stricter security mechanisms. It is possible that in such scenarios, different private 5G networks come from different vendors and are operated by different MNOs or service providers. The standard solution for interoperability and intercommunication is then to establish secure connections between endpoints or between services that are part of the data networks of the enterprises involved, such as VPN connections. Secure external databases or a database hosted by a party can be used to exchange information between enterprises. As in intra-enterprise case, some critical network functions in the control plane must be replicated for reliability and security purposes.

**Fig. 13:**
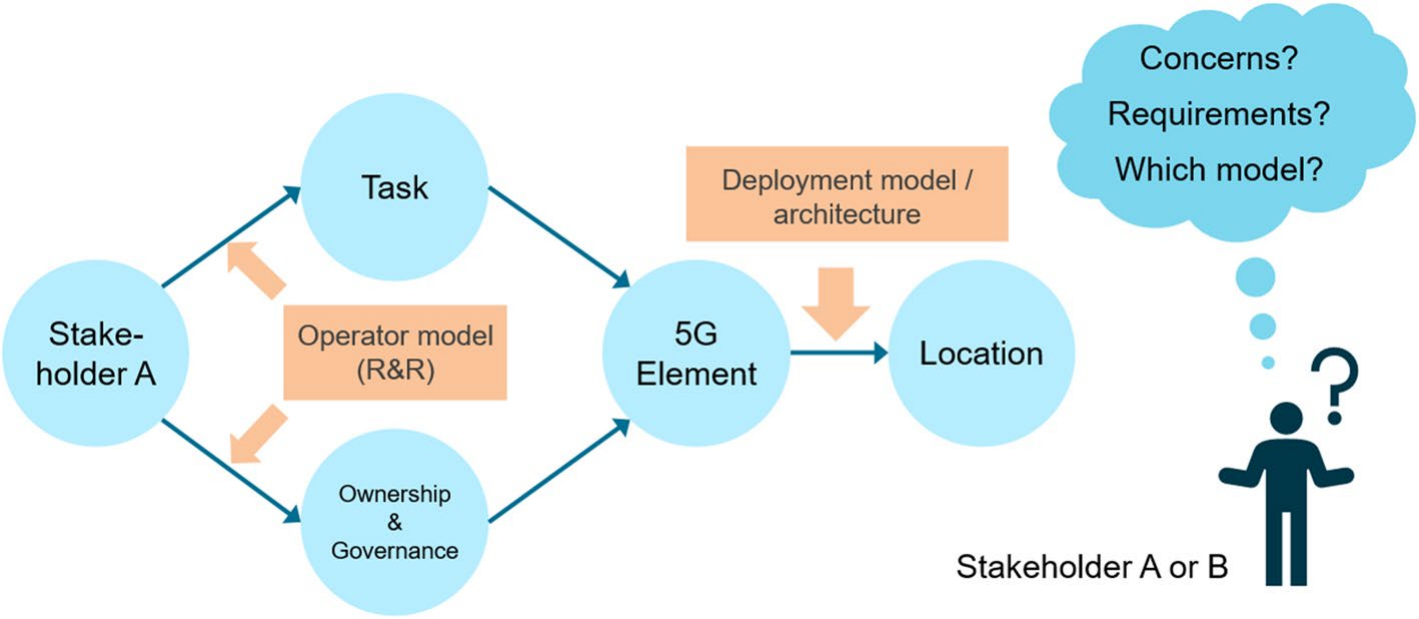
The view of stakeholders on operator models

### Discussions about interdependence between operator and deployment models

Element ownership plays a central role in mapping operator models to the architecture. The simplest case is where the enterprise owns the elements from the user devices to the applications. In this case, the enterprise will have access to the RAN elements, the transport networks, and the core network. In this case, the USIM cards belong to the enterprise. The same is true if the enterprise deploys its own core network and applications, but the RAN is shared and connected to the MNO and enterprise core networks. The USIM cards still belong to the enterprise. But in a hybrid model where the enterprise owns its radio and core network, the enterprise user can access both the MNO network and the private network under certain roaming agreements. In the extreme case, in the MNO’s private network, the enterprise owns its application where the 5G elements and USIM cards are owned by MNO. Depending on the mapping, users must register or subscribe to both the private network and the MNO network or only to a private network. Governance (i.e., management and orchestration) and skill requirements must also be considered when mapping operator models. The enterprise might need specialized telecom engineers to setup and maintain its own elements, while third parties will mainly need a system integration team and the MNO will govern the elements. Some considerations must also be done on spectrum allocation. The private 5G network may use licensed spectrum with the permission of the MNO license owner or government authorized private spectrum or unlicensed band. Before selecting an operator model for an architecture, some KPIs must also be considered. Latency depends on the data path in a certain operator model. In fact, traffic may be sent only to private edge servers or to both the cloud server and the edge server, which may be physically or logically located on-site, in a local data center or in cloud centers. Security considerations are also important and depend heavily on the data path and control path in a certain operator model.

The deployment strategy of private 5G networks may deploy a single operator model or multiple operator models based on coverage and KPI requirements or certain constraints imposed by regulators. For example, the enterprise may deploy a fully private model or a private MNO model. The enterprise can also deploy the MVNO model and the hybrid model at different sites. While some enterprise sites use MNO RAN network for wireless data transmission, some other enterprise sites construct their dedicated RAN network for better wireless coverage or capacity demand. Another example is a global enterprise, which may also deploy both fully private model and MVNO model at different sites. While the enterprise private core network is located at the enterprise headquarters, some branches may not be able to setup their dedicated RAN network regarding the ownership of spectrum or the regulations per country. Therefore, those enterprise branches may deploy a MVNO model. Each 5G-related element can be physically and logically “located” at different systems, platforms or locations. The different locations that could be taken into account for distributing the elements are listed in Table 5. In the following subsections, we consider the relationship to governance and ownership of such 5G-related elements and implications such a placement of a 5G-related element at a location can have on the possible owning and governing stakeholders.

### Discussions from stakeholders’ points of view

The concerns explained in Sect. 4.4 give rise to a number of particular stakeholder requirements that may differ among the individual stakeholders. Each of the four different architecture models presented in this section can address the stakeholders’ requirements in particular way, i.e., they are either inherently fulfilled by the model, by additional technical features, by contractual agreements between at least two stakeholders (if technical features are not available) or they cannot be fulfilled. Figure 13 shows the view of a stakeholder on the problem of choosing a right model given his or her concerns and the requirements derived from these concerns. Some insights with respect to a number of requirement groups and to what extent they are fulfilled by the four different models are explained subsequently.

One group of requirements pertain to **wrong or missing access to elements by a stakeholder**, e.g., remote access to stakeholder’s equipment shall be ensured and the impact of network element failure shall be minimized. In general, fulfilling this group of requirements is less of a problem, when fewer stakeholders are involved in management and operation tasks. For instance, this is the case for the fully private model, where the enterprise retains full control over each and every element, or where the MNO has full control, e.g., in the hybrid and MNO models. Remote access to network elements can be accomplished through standard tools, but if maintenance needs to be carried out locally, access must be guaranteed by the enterprise, which requires bilateral contractual agreements. Additional requirements emerge from the aspect of **interoperability of security systems and alignment of security concepts**. This is of major concern of the party that which wants to integrate the private 5G network into the local IT infrastructure, i.e., the enterprise. One example is that the UDM and encryption keys shall be accessible and governed by the enterprise. In principle, there are no dependencies between MNO or SP security concepts and that of the enterprise in the fully private model. In fact, private 5G can be securely integrated as needed. Obviously, the MNO might prefer the MNO model as this is the one that requires least IT integration efforts. For models, where many different stakeholders are involved, **lack of expertise to carry out certain network lifecycle tasks** might be a main problem. In particular, this applies for the enterprise, which generally might lack competencies regarding cellular network management, which includes handling of the spectrum. Here, the enterprise might prefer an MNO- or SP-operated model, such as the hybrid, MVNO or MNO model. Of course, since MNOs and SPs bring in the right expertise, there might be only a few requirements toward the enterprise depending on tasks that could be transferred to the enterprise. In terms of **confidentiality, integrity and availability of data**, the fully private model may be the clearly preferred model by an enterprise, whereas for the MNO model most requirements can only be fulfilled by contract between the parties, which specify to handle events of data breach, system unavailability, etc., with different kinds of service level agreements. One such requirement is that for confidentiality reasons the UPF shall not be accessible by any other third party in case of unencrypted data transfer. Because fewer other stakeholders are involved in the MNO and hybrid models, they are clearly preferred by an MNO in terms of **stakeholder autonomy**. While QoS guarantees can be given by technical features through the MNO/SP in their view, the enterprise might want to prefer contracts that would also ensure appropriate QoS beyond today’s known use cases. On the contrary, the fully private model might be preferred by the enterprise, where the latter can directly negotiate the network features and QoS guarantees with the network vendor. Other requirements also emerge from concerns related to **ownership of and governance over elements by another stakeholder**, e.g., easy expansion of UE base. While in many cases this might require additional technical features of extension of the wireless network and compute capacity (technical features), the enterprise might require dedicated capacity expansion plans, which are solved through contractual means.

Also related to some of the already mentioned requirements above, further ones belong to reducing coordination effort, multi-site setup support, costs, service availability and continuity as well as global applicability. In summary, all four models have advantages and disadvantages in light of the different requirements of the stakeholders. While not all requirements are inherently fulfilled by the models, most of them can be addressed by additional technical or contractual means. Ultimately, the fully private model might be the one to be considered by (large) enterprises, while MNOs and SPs can quite flexibly apply technical solutions solving most of the challenges of private 5G networks and their operation. Here, the hybrid, MNO and MVNO can play a significant role. Lastly, the actual choice then depends on balancing all the relevant aspects including security, autonomy, costs and global applicability.

## Technological enablers for 5G private networks: methods and results

In order to deploy 5G private network, key enabling technology components as well as optimization and planning methodologies must be discussed or investigated for industrial non-public 5G networks. Before the deployment, stakeholders should consider spectrum selection and license but also channel measurement in order to fully understand the propagation characteristic of the environment and to set up end-to-end system parameters. During the deployment, a monitoring tool is necessary to validate the deployment and to make sure that the end-to-end system meets the target KPI. Finally, some optimization can be made individually for service placement, network slicing, network orchestration or jointly at RAN, MEC or core network level.

### Spectrum allocation and channel models

Private networks constitute a paradigm shift for design and operation of mobile radio networks which originate from standards created for global deployments of public mobile network infrastructure to connect mobile phones everywhere where people live, travel and work. 5G setting the foundation for purpose targeted private networks in particular in an industrial context addressing non-functional and functional requirements and KPIs as well as spectrum and regulation, thus providing the framework for non-public deployments of 5G technology in Mission Critical Communication (MCC) in factory environments.

#### Spectrum allocation models for private networks

The electromagnetic spectrum is, for most parts, regulated by governments and harmonization across regions and continents was key for global success of standardized radio technologies. Some parts of the spectrum are allocated to general purposes, e.g., Industrial, Scientific, Medical (ISM), and are available *unlicensed* under strict usage rules. Other parts of the spectrum are *licensed*, which means that only the license holder can deploy and operate radio equipment and services in this particular spectrum. Spectrum that is designated for terrestrial mobile telecommunication services, needed to operate 4G, 5G and beyond, is usually divided into sub-bands auctioned off to MNO at high prices for a state or country wide license. Such mechanism became an obstacle for availability of designated spectrum to be licensed to professional users for localized private networks which had so far to share unlicensed spectrum potentially with other users and equipment operated in the same spectrum and the same location. This puts feasibility limits for MCC because it is impossible to guarantee quality of service or latency. As a consequence locally available licensed spectrum becomes a prerequisite for the success of Industry 4.0, while unlicensed spectrum provides additional capacity for non-MCC supplementary services. Fortunately, governments have started the process of opening allocated spectrum as licensed shared spectrum or dynamic spectrum sharing for localized use in specific bands to enable the deployment and operation of private 5G networks.

**Licensed Shared Operation:** Several countries, including Germany, UK, and Taiwan, have started the process of allocating parts of the 5G spectrum for local private use instead of for nationwide coverage. Non- internet service providers can apply for a license for up to 100 MHz of spectrum in the range of 3.7 to 4.9 GHz, depending on the country. For a small (yearly) fee, companies can then use those frequencies exclusively on their premises to deploy a private network.

**Dynamic Spectrum Access:** In the USA, the 3.5 GHz frequency band was recently opened up for commercial use by the US Federal Communications Commission. This band is known as the citizen broadband radio service and does not require spectrum licenses. Access and operation is governed by a dynamic spectrum access system, but the users are required to take care not to interfere with others already using nearby bands.

**Fig. 14:**
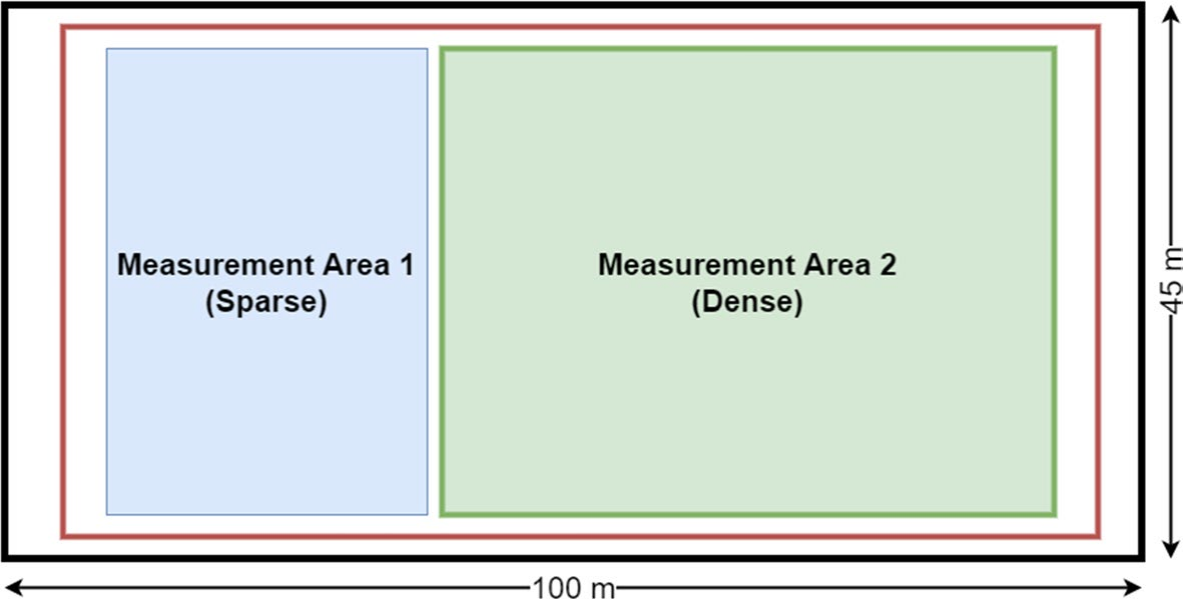
Scenario for the channel measurement campaign

#### Measurement campaign

Before any large-scale system deployment, the propagation characteristics of the environment must be fully understood and parameterized for system level simulations. The aspect of interference coordination is especially important for private networks. Although a first version of the 3GPP TR 38.901 channel model supporting *Indoor-Industrial* scenarios has already been released, further research on the indoor industrial channel [33, 34] is necessary. Due to the highly reflective nature of the environment, caused by shop floors usually densely packed with metallic machinery, the indoor industrial channel shows effects such as diffuse or dense multi-path propagation that are not common in other types of scenarios. During the 5G CONNI project, the consortium conducted an extensive measurement campaign in an industrial environment at 3.7, 28 and 300 GHz. The results of this measurement campaign will be used to contribute to standardization and to enhance existing channel models.

The measurements were conducted in a machine hall with a dimension of approximately 100 by 45 m and a height of 4 m. On the ceiling, metallic air ducts cover most of the hall. Figure 14 shows a stylized floor plan of the machine hall. Several measurements were conducted in two general areas of the hall, displayed in Fig. 14 as Measurement Area 1 in blue and Measurement Area 2 in green. While Measurement Area 1 is sparsely packed with industrial machines and serves as a research, work and storage area, Measurement Area 2 is densely packed with industrial machinery and robots.

In Measurement Area 1, two transmitter locations at a height of 2.7 m above ground were chosen, while the receiver was placed at 15 different locations throughout the area. At both 3.7 and 28 GHz, the measurements were conducted using a *Virtual Uniform Circular Array* [35] antenna in order to estimate the angles of arrival. Additionally, at 9 points, measurements were conducted at 300 GHz [36]. In Measurement Area 2, a single transmitter location was chosen and measurements were conducted at 30 points throughout the area at 3.7 and 28 GHz. At 4 measurement points, the scenario was also characterized at 300 GHz.

In addition to the angle resolved measurements, the large-scale parameters at 3.7 and 28 GHz were also evaluated along a trajectory around the machine hall with a moving receiver. The trajectory is displayed in red in Fig. 14. For these measurements, the transmitter was placed in Measurement Area 1, the receiver was moved at a constant speed of 0.5 m per second along the trajectory, and the channel impulse responses were recorded every 8.1 cm. Finally, *Indoor to Outdoor* measurements were made at 3.7 and 28 GHz.

The results of the measurement campaign will be used both in the 5G CONNI project for connectivity maps and cell planning, and outside of the project for the standardization and refinement of indoor industrial channel models. Initial large-scale parameter evaluations of the measurements at 3.7 and 28 GHz have recently been published in [37].

**Fig. 15:**
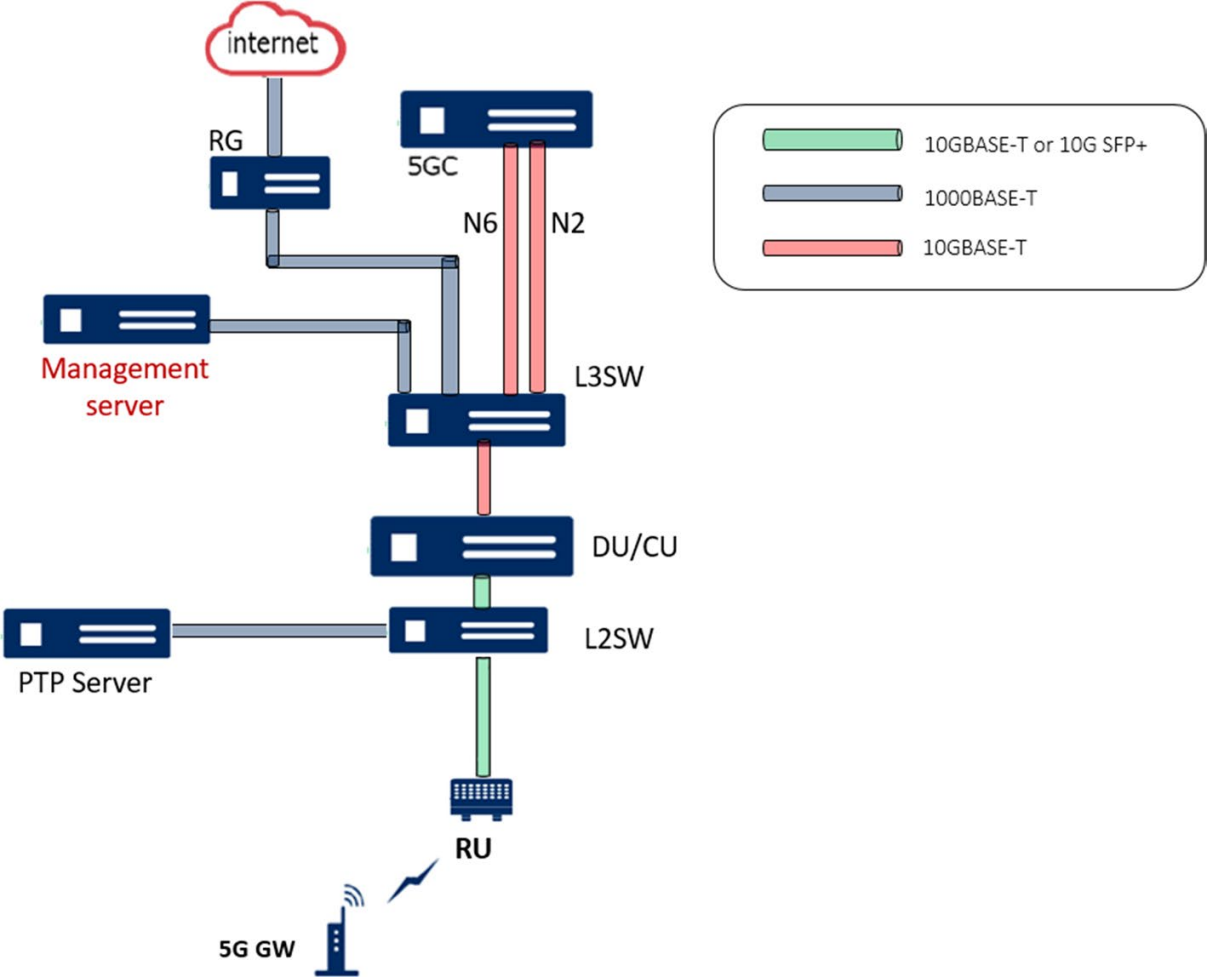
5G end-to-end system

### Monitoring

A 5G end-to-end system including 5G RAN, MEC, CN and related application will be deployed into factories to provide demonstration and service for specific industrial application in 5G CONNI project. The target is not only to build up the 5G enabled communication infrastructure but also to make sure the operation and maintenance of specific industrial application will meet the KPI. To fulfill this, an OAM and KPI monitoring system will be designed into the system as well. Its implementation includes the management of fault, configuration, account, performance and security. In 5G CONNI project, except the account management, other management requirement is going to build up in this 5G end-to-end system. The specific KPI will be implemented with two requirements. One is from 3GPP specification. The other is from the use cases proposed in the project (e.g., end-to-end latency, service bit rate, time synchronization and secure remote access). With the implementation of OAM and associated KPIs in the end-to-end 5G system, users will be able to monitor, configure the system and improve operational efficiency accordingly.

To better describe the implementation, Fig. 15 shows the setup of the end to end 5G system. The management server will configure, e.g., Configuration Management, the RAN that includes the Radio Unit (RU) and Distributed Unite (DU)/ Central Unit (CU) via NetConf protocol while the RAN is setting up. If unexpected scenarios occur, the RAN generates an alarm, e.g., fault management, to the management server. During operation, the RAN generates counters or KPIs to management server. Some KPIs are calculated in the management server to form the KPIs.

### Service placement

In the MEC paradigm, resource-intensive and delay-sensitive applications are handled directly in the edge cloud, avoiding as much as possible the access to cloud computing available in possibly distant data centers to prevent high service delays, which are not tolerable in the Industry 4.0 scenario. Of course the edge cloud offers less storage and computational resources than the large data centers. To overcome this issue, virtualization techniques such as virtual machines and containers help in creating an open edge computing environment, in which storage and computational resources are distributed across the edge cloud when and where needed. To properly exploit this possibility, it is necessary to optimize resource placement and scheduling, in particular, it is necessary to choose the nodes that are the most suitable to store the data and to run the applications offloaded from the querying sensors. In performing this selection, we advocate the use of a joint strategy that considers computational and storage resources jointly. Only few works have considered together communication, computation and caching resource. In [38], the authors solve the problem of service placement and request scheduling aiming to maximize the number of serving requests, allowing direct access only to one server per user. The work in [39] considers overlapping coverage areas for edge nodes with the aim to minimize the request routing to the core cloud (i.e., to maximize the assignment at the edge). Here, we propose a method to minimize the total delay spent on satisfying all service requests, jointly solving the problem where to store services and where to run the requested applications. We consider a wireless edge network consisting of a set ${\mathcal {N}}$ of edge nodes, each one endowed with a MEC server to enable virtualizing network services/applications (VNFs), i.e., equipped with storage, computation and communication capabilities, and endowed with a wireless access point, covering local areas, possibly overlapping. There is a set ${\mathcal {S}}$ of network services, usually consisting of virtual machines and/or containers, able to run such sophisticated applications. Services can be stored on the edge clouds in order to satisfy a set ${\mathcal {K}}$ of sensor devices, each demanding for network services, if there is an active connection link. The aim of our work is to minimize the total system delay occurring for transmitting data from each sensor to an edge server and for processing them. Thus, latency depends on the amount of information data needed to run the desired application, apart from the radio capacity of the transmission link and the computation capacity at the edge cloud. Sensors can offload their data if connected to an access point, then, if none of the neighbor edge nodes cached the demanded service, the device can be routed to a data center, generically indicated as a *core cloud* ${\mathcal {C}}$, storing the entire set *S* of services but placed at a much longer distance. The same occurs if none of the edge server has enough capacity to serve the request. To tackle the problem to solve an Integer Linear Program, we leverage an approximation algorithm based on a randomized rounding [40], with provably guarantees to satisfy the imposed communication and computation constraints on expectation.

**Fig. 16:**
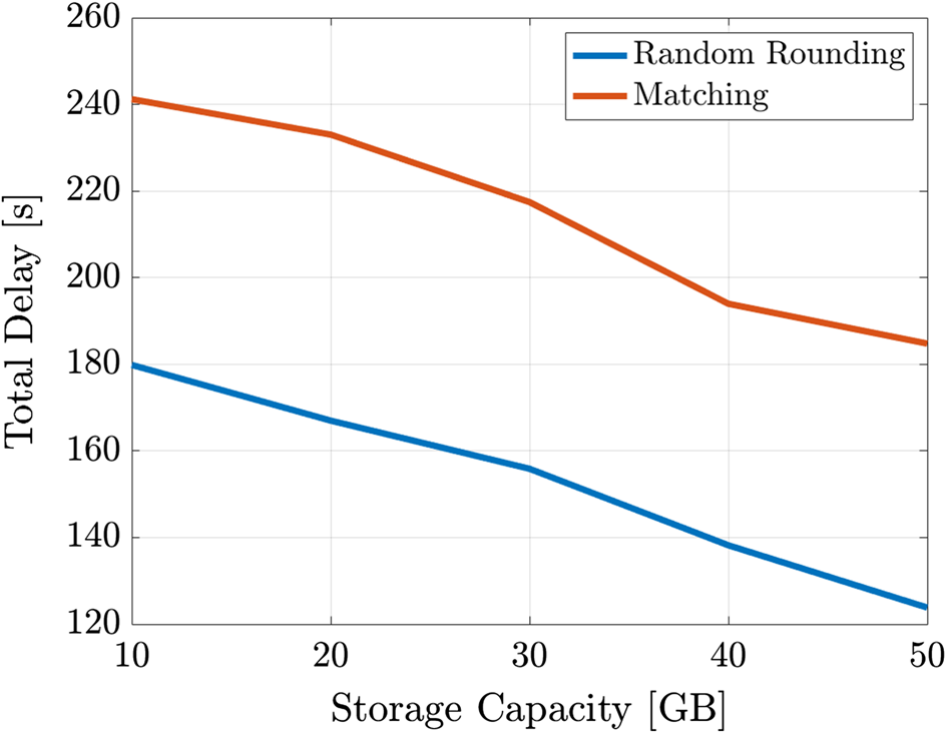
Total delay of network system

To prove the effectiveness of the proposed algorithm, we compare the results with a *matching* algorithm, which aims to route each device to a node with the best SNR on the communication link, an admission occurs if the node has enough storage and computation capabilities to cache and run the requested service. In our simulations, we consider three types of virtual machines [41] micro, small and extra large, with CPU cycles from 500 to 2000 MHz and RAM from 0.6 to 3.7 GB, as processing and storage minimum resource necessary to run the application. The arrivals from sensors are generated with a Poisson distribution with mean arrival rate uniformly distributed $\in [0.4, 1.1]$ Mbps. There are ${\mathcal {K}} = 100$ sensors, uniformly distributed in an area 100 m × 100 m, which offload their data to three possible edge nodes and request services following a popularity profile derived from the Zipf distribution with shape parameter 0.8. The channel model is based on [42]. As we can see from Fig. 16, the matching algorithm experiences a bigger delay due to a frequent request routing to the core cloud, especially when the bound on the storage capacity is very tight.

Our algorithm performs better because the service placement allows the system to amortize the cost of storing a lot of data, reusing the same virtual machine for more users. Of course this can be done for shareable resource as for analytic data cached at the server, otherwise computation and communication resource are dedicated to the specific querying device. The more storage capacity is available at each server, the less requests are routed to the core cloud, and then the system experiences less delay in delivering all the applications. For delay-sensitive services, it is very important to have access at the edge, and the proposed algorithm satisfies the request for such applications in a very fast and efficient way.

**Fig. 17:**
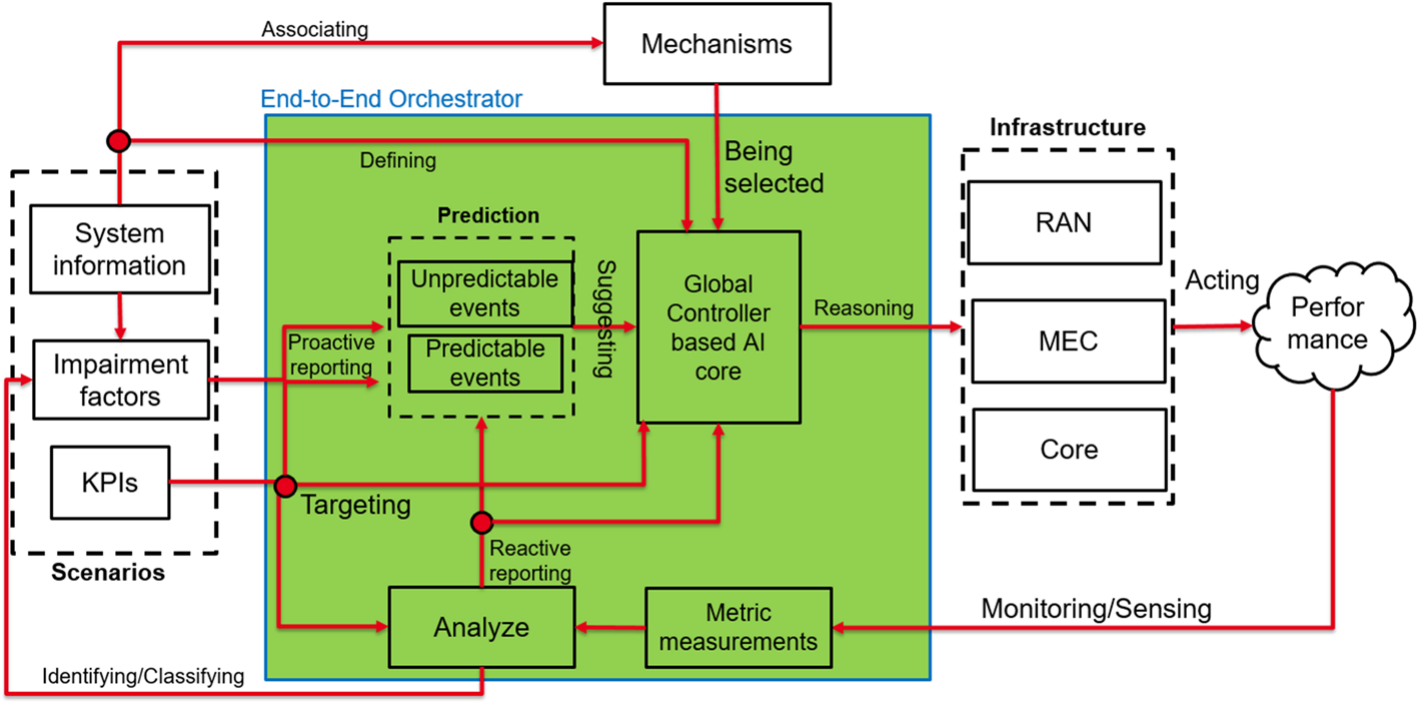
Framework to design a E2E orchestration

### Key enabling technologies

#### MEC

The implementation of Multi-access Edge Computing (MEC) can be based on User Plane Function (UPF) [43] or bump-in-the-wire MEC, which can meet the requirements of high bandwidth and low latency service, and enhance security. In 5G-CONNI project, the UPF for the European testbed is designed according to 3GPP standard, and the MEC platform leverages the 5G network architecture and performs the traffic routing and steering function in the UPF. An UL classifier of the UPF can be used to steer the UP traffic matching the filters controlled by the Session Management Function (SMF) to the local data network, where it can be consumed by the MEC application. The Policy Control Function (PCF) and the SMF can set the policy to influence such traffic routing in the UPF. Also, the application function can influence the traffic routing and steering via the PCF. Therefore, MEC in 5G is able to influence the UPF through the standardized Cloud Provider (CP) interface in the SMF. Furthermore, the MEC platform is completely virtualized environment in order to enable seamless application lifecycle management paired with seamless platform management. On the other hand, the bump-in-the-wire MEC for the Taiwanese testbed is implemented according to ETSI standard, where MEC cloud includes an NFV infrastructure and data plane functions. NFV infrastructure is named ECoreCloud. The data plane functions comprise Software-Defined Networking (SDN) switch and Mobile Edge Enabler (MEE) VNF developed, where SDN switch is used to route and mirror the traffic, and MEE VNF provides a traffic steering function so that selected data traffic can be offloaded locally. The MEE VNF is divided into two modules, including the Control Plane Analyzer module and Data Plane Processor. The Control Plane Analyzer module takes charging in decoding and correlating signals, and the Data Plane Processor is to process data plane traffic and steering. The bump-in-the-wire MEC standalone prototype is deployed between 5G New Radio and 5G standalone core network, which is a convenient deployment because it does not need the additional configurations for the core and RAN network. Furthermore, the MEC must handle N2 interface according to 3GPP TS38.413 [44] and process the GPRS tunneling protocol user plane extension header packet for N3 interface based on 3GPP TS29.281 [45] so that the GPRS tunneling protocol user plane can be handled properly.

#### Network slicing and orchestration

Network slicing is a key feature of 5G networks to support diverse requirements on a single physical infrastructure through multiple logical virtual network functions. The virtual network functions are managed by NFV technology, which flexibly allocates virtual resources and provides modular architecture. In addition, SDN enables the communication between virtual network functions. By programmable network routing and separation planes, SDN technology achieves resource isolation for each network slice. With the benefit of NFV and SDN, network slicing allows operators to fast create on-demand network services for 5G vertical industries (e.g., smart factories, remote robotic surgery, and autonomous driving) based on slice configuration. Our goal is to realize a NFV-like lightweight 5G core in 5G CONNI. First, we implement a set of VNFs to run a 4G core and analyze the total completion time including instantiation time and configuration time as our performance metric. Based on our experimental result, the completion time is less than 12 minutes for deploying a total of 20 instances. The deployment efficiency is acceptable for operators to fast create different services. As a result, we will extend the lightweight framework with the same architecture to run a 5G core prototype including AMF, SMF, AUSF, UDM, and UPF.

Network orchestration is another key feature of 5G networks. The orchestrator needs to efficiently manage the 5G network in order to meet diverse and/or extreme QoS requirements and AI can play a key role in providing sub-optimal approaches. Because of the diversity and variation over time of QoS requirements, a versatile and adaptable network is needed to configure network parameters in dynamic environments and contexts while maintaining performance. Network management must also elastically orchestrate RAN, MEC, transport and core networks simultaneously, by exploiting scalable and flexible infrastructure.

In 5G CONNI, we propose an original methodology to design an end-to-end orchestrator taking into account the heterogeneity and coexistence of services, the dynamic evolution of needs (e.g., traffic, number of users, QoS) and the changing environment. The AI-based end-to-end orchestrator measures, predicts the network performance, dynamically modifies network parameters and elastically combines diversity to face to a multitude of (un)predictable impairments. Its design dynamically, elastically and efficiently deals with the complex ecosystem of tenants, network slices with a diversity of service requirements and efficient usage of resources. Figure 17 illustrates the orchestrator framework.

**Fig. 18:**
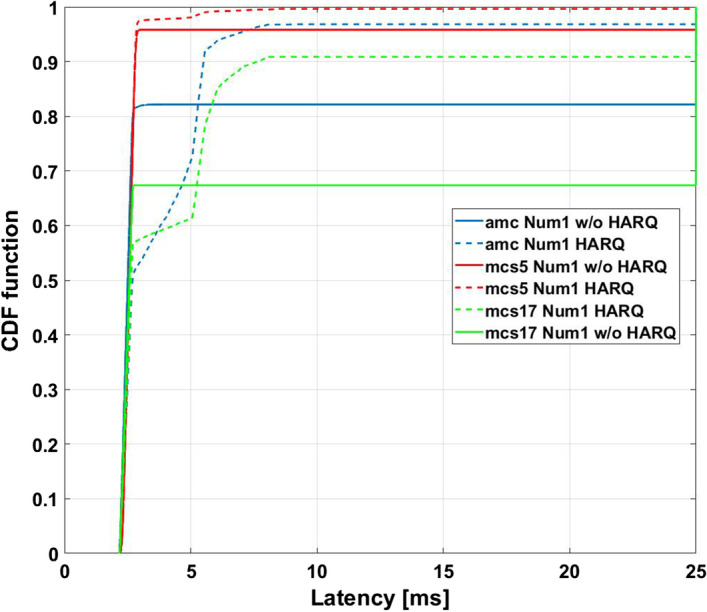
CDF of E2E latency for MCS5, MCS17 and AMC

Before evaluating the AI-based orchestrator, we have modified a 5G NR network simulator based on NS-3 to multiplex low latency mechanisms (e.g., frame design) and reliability enhancing mechanisms (e.g., multiple antenna, redundancy and adaptive Modulation and Coding Scheme (MCS) exploiting code/time/space diversity). This simulator is used to evaluate the impact of the combination of mechanisms exploiting a diversity subset on the end-to-end Ultra-Reliably Low-Latency Communications (URLLC) performance at RAN level cooperating with EPC/LTE core network. Figure 18 compares the performance of the Adaptive Modulation and Coding (AMC) mechanism with the robust MCS5 and the high throughput MCS17 in a fast fading channel for indoor office scenario. The results provide insights into the behavior of different combinations with respect to URLLC performance and are a first step for further research using AI.

**Fig. 19:**
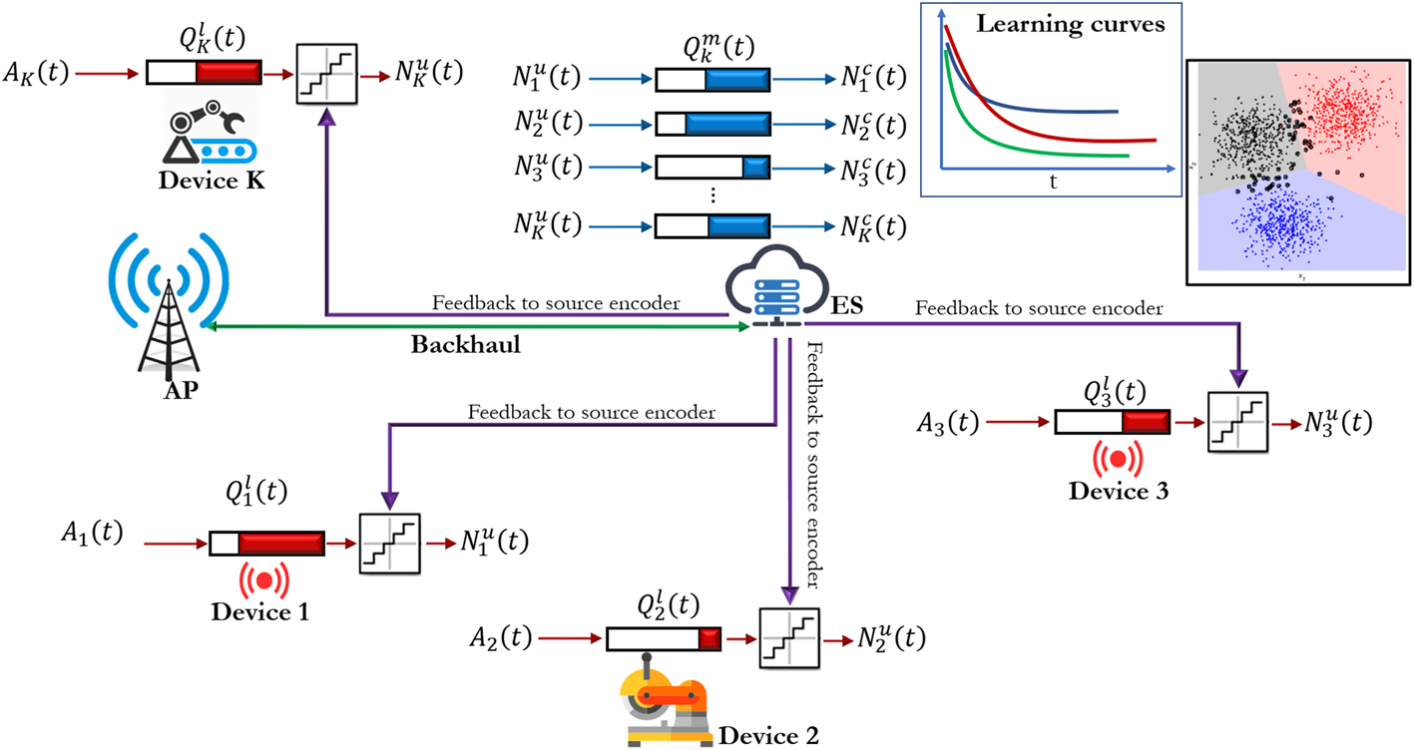
Edge machine learning scenario [46]

#### Core network

In the Taiwanese testbed, the 5G CN is designed based on service-based architecture and follows 3GPP Release 15+ as a standalone solution. The III-5G CN containerizes all core network functions with Control/User split architecture, enabling the enterprise to distribute these functions wherever and whenever needed. All the modules can be deployed on virtual machines on top of a large number of virtualization environments, and managed as a Kubernetes platform. The AMF establishes the UE context and Packet Data Unit (PDU) resource allocation via Single Network Slice Selection Assistance Information (S-NSSAI) provided by the UE. The S-NSSAI is set up per PDU session for the policy management in the PDU session level. The SMF controls the user plane function and therefore directs and redirects the service flows as required for the applications. We are testing the core network basic functions like UE registration, PDU session establishment, service request and Xn & N2 handover procedure via Spirent Landslide emulator. Furthermore, for supporting the industrial applications, the development especially focuses on data plane efficiency and system reliability. Thus, we are developing both software and hardware accelerating solutions for data plane to enhance packet processing and load monitoring.

#### Joint optimization of enabling technologies

In private industrial networks, devising computation offloading strategies to enable complex processing of data collected by mobile inspectors/sensors/machines is necessary in order to guarantee continuous monitoring and anomaly detection and control decisions during industrial processes. Thus, we now present a strategy for dynamic resource allocation for computation offloading of machine learning tasks at the edge, in the new framework of *Edge Machine Learning* [46, 47]. The goal is to allocate radio (e.g., transmit power, quantization) and computation resources (e.g., CPU scheduling at the edge server), to explore the trade-off between network energy consumption, end-to-end (E2E) delay and accuracy of the learning task. Here, the E2E delay is intended as the time elapsed from the generation of a new data unit by a sensor/mobile device, until its computation is performed at the Edge Server (ES) to run the online learning task. Indeed, as clear from Fig. 19, data units experience a local queueing delay (red queue), a transmission delay for data uploading to the ES, a remote queueing delay at the ES (blue queue), and a computing time to be elaborated. The output of the computation performed by the ES is an estimation/prediction/classification result (up right part of the figure).

In [48], the authors present a distributed learning algorithm at the edge, where end devices, helped by an ES, minimize an empirical loss function in a collaborative fashion. The work in [49] proposes an strategy to maximize learning accuracy under latency constraints. Elgabli et al. [50] proposes a decentralized machine learning algorithm that dynamically optimizes a stochastic quantization method, with applications to regression and image classification, and with a communication-efficient perspective. A stochastic gradient descent distributed machine learning algorithm at the edge is presented in [51]. In particular, the authors consider the trade-off between local update and global aggregation. In [52], the authors present a data compression algorithm to reduce the communication burden and energy consumption of an IoT network, to enable machine learning with a desired target accuracy. Edge machine learning is a research topic at its infancy, so that the research community just started investigating the possible directions. Our goal is to *jointly* optimize radio and computation resources to minimize the network energy consumption, under constraints on the E2E delay and the accuracy of the learning tasks, which in this case is an estimation task based on least mean squares. The power of the method is that it does not require any prior knowledge of the statistics of data arrivals, radio channels, and data distributions. It should be noted that both the transmit energy consumption of the sensors and the learning accuracy are affected by the number of quantization bits used to encode the data. More bits lead to better accuracy but higher energy consumption, due to the longer payloads to be transmitted. As a measure for the accuracy, we use the Mean Squared Deviation (MSD) between the true parameter and the estimation performed on offloaded data. More technical details can be found in [46].

**Fig. 20:**
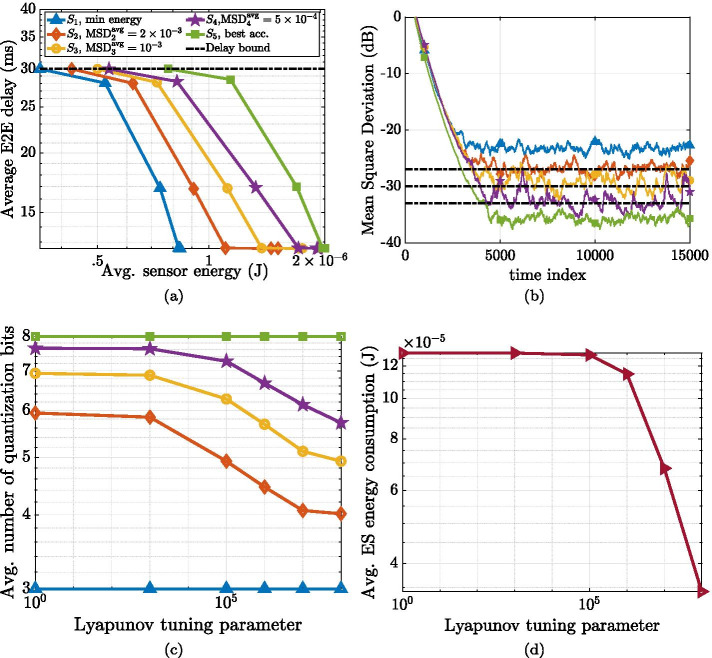
Energy-delay-accuracy trade-off [46]

We now illustrate the performance of our solution in terms of trade-off between energy, delay, and learning accuracy. In particular, we want to highlight how, given a certain E2E delay constraint (set to 30 ms), the accuracy affects the performance in term of energy consumption. To this aim, we consider 5 sensors at the same distance from the AP, with the same arrival rate, the same average E2E delay constraint, but different constraints on the MSD (i.e., the accuracy). In particular, we assume that two sensors represent two benchmarks: (i) minimum energy, obtained by the device always transmitting with 3 quantization bits (i.e., the minimum number of bits), for all t; (ii) best accuracy, obtained by the device always transmitting data with 8 quantization bits (i.e., the maximum number of bits). The other three devices have different intermediate requirements for the MSD. In Fig. 20a, we show the average E2E delay as a function of the sensor energy consumption, obtained by tuning a trade-off parameter from Lyapunov optimization [46]. In particular, the parameter increases from right to left, as shown in the figure. From Fig. 20a, we can notice how the energy consumption decreases while the E2E delay increases. However, this trade-off is different among the different devices due to the different accuracy constraints. In particular, let us first comment on the results for the two benchmarks. The green curve (squared marker) shows the best accuracy case, which indeed achieves the highest minimum energy among all devices, given the E2E delay (the legend of all figures is shown in Fig. 20a). At the same time, from Fig. 20b, which shows the MSD vs. the time index, we can notice how this device achieves the minimum MSD. These first two results are due to the maximum number of quantization bits shown in Fig. 20c, which reaches the highest value for this device. Note that the average number of quantization bits is shown as a function of the Lyapunov tuning parameter, using the same values as for Fig. 20a. On the other hand, the blue curve (triangle marker) represents the minimum energy case, and it achieves the worst accuracy due to the minimum number of quantization bits adopted, as can be noticed from Fig. 20a–c. The other curves represent intermediate energy cases obtained fixing a target MSD constraint, and can be interpreted via similar analysis over Fig. 20a–c. Finally, the energy consumption of the ES decreases as the Lyapunov parameter increases, until reaching a floor as the device energy consumption (Fig. 20d). In summary, the take-home message of Fig. 20 is twofold: (i) Our method is able to obtain a low system energy solution with accuracy and E2E delay guarantees; (ii) by relaxing the accuracy constraint, lower energy can be achieved due to the lower number of quantization bits, which translates into a lower average data rate over the wireless interface.

The accuracy required is highly dependent on the particular use case. It is not always desirable to obtain the best possible accuracy, if it results in higher energy cost, given an E2E delay. With our method, by setting a target accuracy, it is possible to achieve lower energy consumption without degrading the application performance below the target threshold.

## Conclusions

In this paper, we presented the vision and initial results of the joint Europe-Taiwan H2020 5G CONNI project. The research focuses on non-public 5G networks in Industry 4.0 applications, ultimately aiming to deploy an end-to-end 5G test system for key enabling technologies ranging from 5G Core, MEC and 5G RAN to the industrial use cases. For the 5G CONNI demonstration system, three challenging industrial manufacturing use cases are presented along with a number of multi-site connectivity scenarios.

Since non-public networks lead to disaggregation of network elements not only at the technical level but also at the organizational level, possible deployment and operator models need to be considered. We present an in-depth conceptual discussion of their interdependence and a detailed evaluation of operator models based on four new network architectures for non-private deployments.

Finally, we presented results of work toward the end-to-end system, including key enabling technology components as well as optimization and planning methodologies specifically targeting 5G non-public industrial networks.

## Data Availability

Not applicable.

## Abbreviations

3EC: Third-party Enterprise/Community
3GPP: 3rd Generation Partnership Project
3NP: Third-party Network/radio Planner
3SI: Third-party System Integrator
3WO: Third-party WAN Operator
5G: fifth generation of mobile communication networks
5G ACIA: 5G Alliance for Connected Industries and Automation
5G CONNI: 5G for Connected Industries
AGV: Automated Guided Vehicle
AI: Artificial Intelligence
AMC: Adaptive Modulation and Coding
AMF: Access and Mobility Management Function
AR: Augmented Reality
AUSF: Authentication Server Function
BM: Business Model
CN: Core Network
CNC: Computer Numerical Control
CP: Cloud Provider
CPE: Customer Premise Equipment
CPU: Central Processing Unit
CSP: Communication Service Provider
CU: Central Unit
DECOR: Dedicated Core Network
DU: Distributed Unit
E2E: End-to-End
EPC: Evolved Packet Core
ES: Edge Server
eSIM: Embedded-SIM
ETSI: European Telecommunications Standards Institute
gNB: gNodeB
IEC: International Electrotechnical Commission
IoT: Internet of Things
IP: Internet Protocol
ISA: International Society of Automation
IT: Information Technology
I-UPF: Intermediate-UPF
KPI: Key Performance Indicator
MCC: Mission Critical Communication
MCS: Modulation and Coding Scheme
MEC: Multi-Access Edge Computing
MEE: Mobile Edge Enabler
MNO: Mobile Network Operator
MOCN: Multi-Operator Core Network
MSD: Mean Squared Deviation
MVNO: Mobile Virtual Network Operator
NEF: Network Exposure Function
NEV: Network Equipment Vendor
NFV: Network Function Virtualization
NG-RAN: New Generation RAN
NPN: Non-Public Network
OAM: Operation, Administration and Management
PC: Personal Computer
PCF: Policy Control Function
PDU: Packet Data Unit
PLMN: Public Land Mobile Network
QoS: Quality of Service
RAN: Radio Access Network
RRC: Radio Resource Control
RU: Radio Unit
SDN: Software-Defined Networking
SIM: Subscriber Identity Module
SMF: Session Management Function
SP: Service Provider
TN: Transport Network
T&E: Trial and Error
UC: Use Case
UDM: Unified Data Management
UDP: User Datagram Protocol
UE: User Equipment
UPF: User Plane Function
URLLC: Ultra-Reliably Low-Latency Communications
VNF: Virtualized Network Function
VO: Virtual Operator
VR: Virtual Reality
WAN: Wide Area Network
